# Facilitators and Barriers to Digital Health Technologies for Self-Management in Patients With Chronic Kidney Disease: Systematic Review Based on the Updated CFIR 2.0 Framework

**DOI:** 10.2196/103560

**Published:** 2026-09-21

**Authors:** Feiyue Su, Yao Yao, Xiaoqi Lin, Qingwen Su, Yichao Hu, Hualian Pei, Qinhong Xu

**Affiliations:** 1 Department of Nursing, The First Affiliated Hospital of Ningbo University, 59 Liuting Rd, Haishu District, Ningbo, 315000, China, +86 87085014; 2 Department of Urology, The First Affiliated Hospital of Ningbo University, Ningbo, China

**Keywords:** chronic kidney disease, digital health technology, self-management, consolidated framework for implementation research, systematic review

## Abstract

**Background:**

The integration of digital health technology (DHT) into chronic kidney disease (CKD) care holds transformative potential for enhancing patient self-management and slowing disease progression. Despite the growing availability of DHT, there remains limited understanding of the factors that facilitate or hinder their adoption and use among patients with CKD.

**Objective:**

This study aims to identify the facilitators and barriers to the use of digital health interventions for self-management in patients with CKD, and to provide evidence to inform the development of implementation strategies.

**Methods:**

A systematic search was performed across 11 databases (CNKI, WanFang Data, VIP Database, Chinese Medical Journals Database, PubMed, CINAHL, Embase, Cochrane Library, Scopus, PsycINFO, and Web of Science Core Collection) from database inception to May 12, 2026, with reports restricted to those published in English or Chinese. Eligible studies were required to enroll adult patients with CKD (aged ≥18 years); report facilitators, barriers, or influencing factors related to DHT use for self-management; and use qualitative, quantitative, or mixed methods designs. Nonempirical articles, conference abstracts, and studies without accessible full text were excluded. Two researchers (Feiyue Su and Yao Yao) independently assessed the quality of the included studies using the Mixed Methods Appraisal Tool (MMAT). Extracted data were coded deductively to the Consolidated Framework for Implementation Research 2.0 (CFIR 2.0) constructs using open and axial coding; recurring themes were then synthesized into a narrative summary organized by the 5 CFIR domains.

**Results:**

Thirteen studies (8 mixed methods, 3 quantitative, and 2 qualitative) were included, encompassing 3002 patients with CKD across 8 countries. Of these, 9 met all applicable MMAT criteria, while the remaining 4 had methodological limitations. In total, 19 facilitators and 15 barriers were identified and mapped to the 5 CFIR 2.0 domains. The most frequently reported facilitators were a simple and easy-to-use user interface (9 studies, high confidence), care and support from family members and peers (8 studies, high confidence), convenient anytime access to health information, high perceived usefulness, personalized educational content, and continuous content updates (each reported in 5 studies, high confidence). The most frequently reported barriers were low health literacy (6 studies, high confidence), poor economic status of patients (6 studies, moderate confidence), and advanced age (5 studies, high confidence).

**Conclusions:**

The use of digital health interventions for CKD self-management is influenced by multilevel factors. However, the evidence base is limited by the predominance of mixed methods designs, the lack of longitudinal studies, and the concentration of studies in high-income countries, which may limit the generalizability of the findings. Targeted, multilevel strategies that address the identified facilitators and barriers are essential for enhancing effective and equitable implementation.

## Introduction

### Background

Chronic kidney disease (CKD) is a complex condition characterized by a progressive decline in kidney function. It is associated with high prevalence, an elevated risk of complications, long-term treatment requirements, and substantial health care costs [1]. Among all chronic diseases, CKD has the highest disability and mortality rates and is projected to become the fifth leading cause of death worldwide by 2040 [2]. Reducing the economic and health burden of CKD is therefore a critical global public health priority.

Self-management in chronic disease is defined as “the process by which patients actively adopt healthy behaviors, systematically monitor and manage disease-related symptoms, and thereby mitigate the negative impact of the disease on physiological function, psychological status, social functioning, and interpersonal relationships” [3,4]. Research has shown that effective self-management practices not only alleviate clinical symptoms and adverse drug reactions but also significantly reduce complication rates, mortality, and hospital readmission rates. In addition, self-management reduces patients’ economic burden, promotes physical and mental health recovery, and ultimately improves overall quality of life [5]. In current clinical practice, the disease management model for CKD has gradually shifted towards a patient-centered self-management approach [6,7]. However, despite the well-recognized benefits of self-management, inadequate implementation of self-management interventions often leads to poor outcomes [8].

Technological advances have fostered the adoption of new digital health technology (DHT). DHT is an umbrella term that encompasses smartphone apps, wearables, online platforms, and other digital solutions designed to promote health and well-being or enhance the efficiency and quality of health and social services [9]. These technologies offer a new approach for patients with CKD to engage in self-management and delay disease progression. Continuity of care delivered via digital health interventions can transcend geographical and temporal barriers, providing patients with ongoing, professional support.

Previous systematic reviews have examined digital health interventions for CKD self-management, but they have predominantly focused on clinical effectiveness and intervention outcomes rather than on the contextual determinants of implementation [8,10-12]. One review limited its scope to dietary mobile apps and nutritional indicators [10]; another synthesized quantitative and qualitative evidence on effectiveness, but did not use a standardized implementation framework such as Consolidated Framework for Implementation Research 2.0 (CFIR 2.0) [8]; and the two most recent reviews narrowed their focus to medication adherence [11] or psychological health, self-efficacy, and quality of life [12]. None of these reviews systematically identified and categorized the multilevel factors that influence patients’ adoption and sustained use of DHT.

### Objective

This systematic review aims to identify and categorize the facilitators and barriers to digital health interventions for self-management in patients with CKD using the updated CFIR 2.0 [13], mapping these factors to the 5 CFIR domains (innovation, outer setting, inner setting, individual characteristics, and implementation process). The review also aims to provide an evidence-based reference for developing targeted strategies to enhance the implementation of digital health-assisted self-management in CKD care.

## Methods

### Research Design

This systematic review was conducted and reported in accordance with the PRISMA (Preferred Reporting Items for Systematic Reviews and Meta-Analysis) guidelines [14] (Checklist 1). A mixed methods systematic review (MMSR) approach was used to integrate evidence from quantitative, qualitative, and mixed methods studies, thereby accommodating the methodological diversity of eligible literature [15]. CFIR is a widely used implementation framework. Although the original 2009 version has been extensively applied, it has recognized limitations, including construct overlap, complexity, and a lack of team process elements [13,16]. The updated CFIR 2.0 offers clearer definitions, reduced overlap, and improved applicability, making it more suitable for multilevel implementation analysis. The updated CFIR 2.0 was therefore adopted to guide the data synthesis and interpretation process in this review. This systematic review has been registered with PROSPERO (International Prospective Register of Systematic Reviews; CRD420261394369).

### Research Question

The research question was structured using the Population, Interest of phenomenon, Context, and Study design (PICOS) framework. The target population (P) comprised adult patients with CKD aged 18 years or older. The interest of phenomenon (I) was barriers, facilitators, or influencing factors related to the use of digital health interventions for self-management. The context (Co) was patients’ use of digital health interventions for self-management. The study designs (S) included qualitative, quantitative, and mixed methods studies.

### Inclusion Criteria

Studies were included if they: (a) enrolled adult patients with CKD (aged ≥18 years); (b) reported facilitators, barriers, or influencing factors related to the use of digital health interventions for self-management; (c) employed qualitative, quantitative, or mixed methods designs.

### Exclusion Criteria

Studies were excluded if they: (a) lacked accessible full text (eg, abstracts, conference papers, and unpublished literature), even after contacting the corresponding authors; (b) were duplicate publications or contained overlapping data; (c) were not written in English or Chinese; or (d) were nonempirical articles such as reviews, commentaries, or theoretical discussions.

### Search Strategy

The search strategy was evaluated by an independent reviewer (Hualian Pei) using the Peer Review of Electronic Search Strategies (PRESS) checklist [17]. First, a preliminary search was conducted in PubMed, and the reviewer briefly analyzed the results to confirm the appropriateness of the strategy. Subsequently, two researchers (Feiyue Su and Yao Yao) performed systematic searches across Chinese databases (CNKI, WanFang, VIP Database, and Chinese Medical Journals Database) and English-language databases (PubMed, CINAHL, Embase, Cochrane Library, Scopus, PsycINFO, and Web of Science), using a combination of MeSH and free-text terms (Table 1 for a conceptual summary of the search terms; Multimedia Appendix 1 for the complete search strategies). The search was conducted from database inception to 12 May 2026.

**Table 1.** MeSH^a^ and free-text terms for identifying facilitators and barriers to DHT-assisted^b^ self-management in patients with CKD^c^.

	Population	Interest of phenomenon	Context	Study designs
MeSH term	“Renal Insufficiency, Chronic” OR “Kidney Failure, Chronic” OR “Renal Dialysis”		(“Telemedicine” OR “Mobile Applications” OR “Smartphone” OR “Computers, Handheld” OR “Internet”) AND (“Self-Management” OR “Self Care”)	“Qualitative Research” OR “Cross-Sectional Studies” OR “Randomized Controlled Trial” OR “Controlled Clinical Trial”
Free terms	“Chronic kidney disease” OR “CKD” OR “Renal insufficiency” OR “Kidney disease” OR “Dialysis” OR “Hemodialysis” OR “Haemodialysis” OR “End stage renal disease” OR “ESRD^d^”	“Barrier” OR “Facilitator” OR “Challenge*” OR “Enabler” OR “Promote” OR “Drive” OR “Experience” OR “Perception” OR “Perspective” OR “Attitude” OR “View” OR “Obstacle” OR “Encourage” OR “Hinder” OR “Discourage”	(“mHealth^e^” OR “Mobile health” OR “eHealth” OR “Telehealth” OR “Mobile app*” OR “Smartphone” OR “Health app*” OR “Web-based” OR “Internet” OR “Digital” OR “Digital health”) AND (“Self-manag*” OR “Self-care” OR “Self monitor*” OR “Self efficacy”)	“Qualitative” OR “Cross-sectional” OR “Cross sectional” OR “Mixed method” OR “Mixed methods” OR “Multimethod” OR “Quantitative and qualitative” OR “Qualitative and quantitative” OR “Randomized controlled trial” OR “RCT^f^” OR “Prospective” OR “Cohort” OR “Longitudinal”

a MeSH: Medical Subject Headings.

b DHT: digital health technology.

c CKD: chronic kidney disease.

d ESRD: end stage renal disease.

e mHealth: mobile health.

f RCT: randomized controlled trial.

### Studies Screening

All retrieved records were imported into EndNote (Clarivate Analytics) for duplicate removal. Two reviewers (Feiyue Su and Yao Yao) independently screened the titles and abstracts of all remaining records against the predefined research questions and exclusion criteria. Full texts of records passing the initial screening were then independently assessed by the same two reviewers. Prior to formal screening, a calibration exercise was conducted on a random subset of 25 records to align the reviewers’ understanding of the eligibility criteria. Records classified as “uncertain” by either reviewer were reexamined jointly. Any disagreement was resolved through discussion; when consensus could not be reached, a third researcher (Xiaoqi Lin) was consulted to make the final decision on inclusion.

### Studies Quality Evaluation

The methodological quality of the included studies was assessed using the Mixed Methods Appraisal Tool (MMAT), a validated instrument suitable for evaluating diverse study designs [18]. In accordance with MMAT guidelines, quality was assessed at the individual criterion level rather than through summary scores, and studies were not excluded on the basis of low methodological quality. When the two researchers (Feiyue Su and Yao Yao) disagreed and could not reach consensus through discussion, a third reviewer (Xiaoqi Lin) was consulted, and the final decision was reached after discussion within the research group.

### Data Extraction

From each included study, we extracted the following information: publication year, authors, country, recruitment setting, sample size, age, study design, and data collection methods. For qualitative studies, we additionally extracted thematic frameworks, descriptions of participant experiences, and reported facilitators and barriers. For quantitative studies, we extracted outcome measure definitions, statistical analyses, and narrative summaries of results. For mixed methods studies, qualitative and quantitative data were extracted separately.

### Data Synthesis

A convergent integrated approach was used for the mixed methods synthesis, following Joanna Briggs Institute (JBI) guidance. Quantitative findings were transformed into textual narrative descriptions through qualitization and then integrated with qualitative themes for combined synthesis. Two researchers (Feiyue Su and Qingwen Su) jointly coded all synthesized thematic statements to CFIR 2.0 constructs using a deductive approach; statements that could not be mapped to a specific construct were assigned to the most relevant domain. A third researcher (Yichao Hu) was consulted to resolve any disagreements. All coding decisions were documented and reviewed iteratively to ensure consistency.

### Assessment of Confidence in Findings

The Grading of Recommendations Assessment, Development and Evaluations-Confidence in Evidence from Reviews of Qualitative Research (GRADE-CERQual) approach was applied to assess confidence in review findings. Four components were evaluated: (1) methodological limitations of contributing studies; (2) coherence of findings; (3) adequacy of data; and (4) relevance to the review question. Each finding was assigned a confidence rating (high, moderate, or low) based on the combined assessment of these components. Detailed assessments are presented in Multimedia Appendix 2.

## Results

### Studies Selection

**Figure 1.**
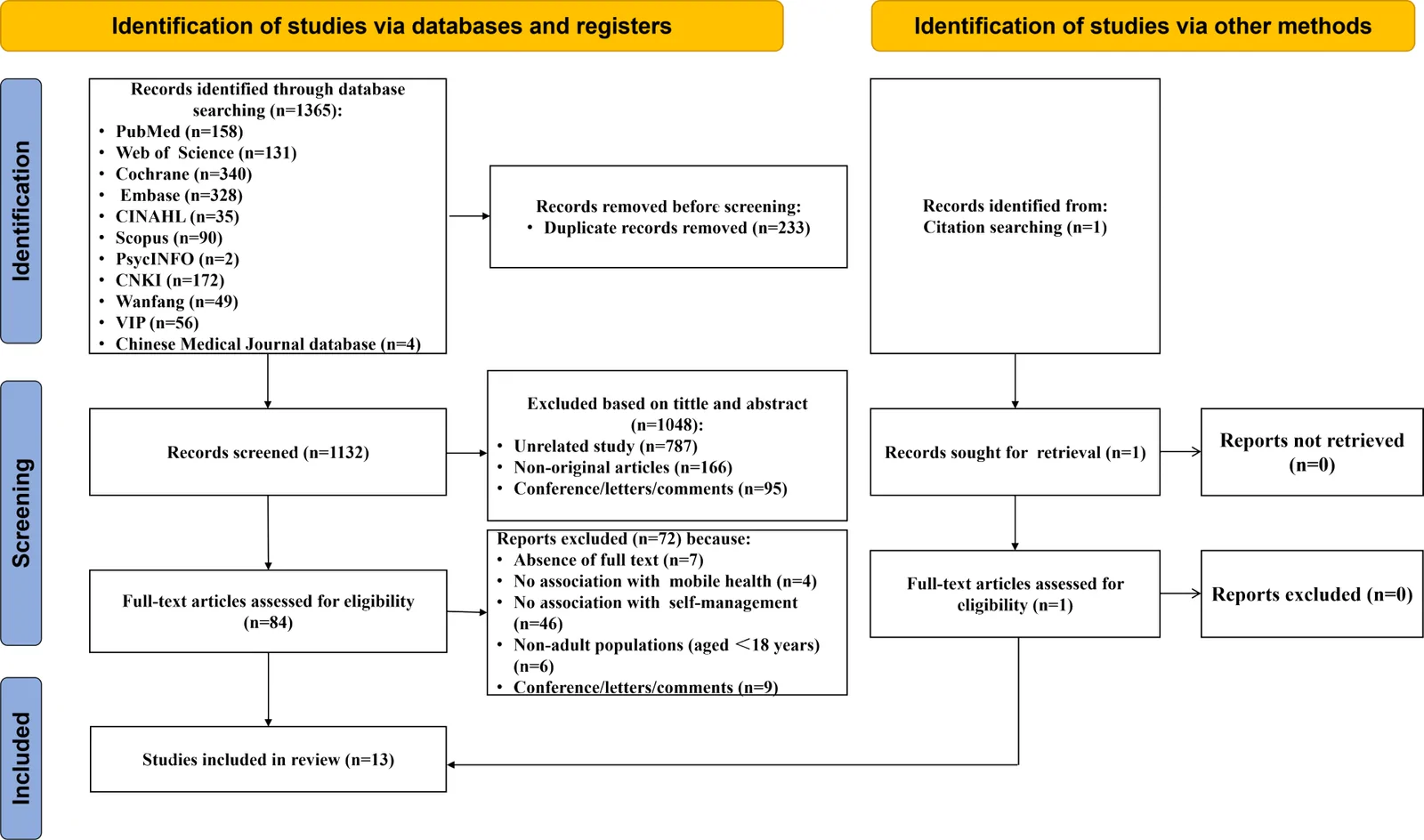
Selection process of the eligible studies.

Two researchers (Feiyue Su and Yao Yao) initially identified 1365 records based on the search strategy. After removing duplicates, 1132 records were screened based on title and abstract, of which 1048 were excluded due to being unrelated studies, non-original articles, or conference letters or comments. After full-text review, 72 irrelevant studies were excluded, while one additional study was included through citation searching. Ultimately, 13 eligible studies were included [19-31] (Figure 1 for details).

### Characteristics of the Eligible Studies

Table 2 presents the detailed characteristics of the 13 eligible studies. The included studies were published between 2018 and 2025 across eight countries or regions, with the highest number of publications occurring in 2022 (n=4). Most studies were conducted in the United Kingdom (n=3). Of the 13 studies, eight used a mixed methods design, three were quantitative (including two cross-sectional studies and one prospective interventional study), and two were qualitative, with semistructured interviews as the primary data collection method. The sample sizes ranged from 11 to 932 participants.

**Table 2.** Characteristics of the eligible studies (n=13). For Donald et al (2022) and Bonner et al (2018), mean age was estimated from the reported age group distributions using standard weighted estimation methods, as the primary studies did not report these values directly.

Study	Country or region	Aim	Recruiting institutions	Study design	Sampling method	Sample size	Age, mean (SD) years	Data collection methods
Lightfoot et al, 2025 [19]	United Kingdom	To explore the uptake and usability of “My Kidneys & Me” (MK&M) alongside patient experiences of using MK&M.	26 hospitals	Mixed methods research	Quantitative: randomization (2:1 allocation, stratified by age ≤63 years and >63 years Qualitative: purposive sampling	420	59.4 (13.6)	Quantitative: perceived usefulness survey Qualitative: semistructured interviews, think-aloud
Sousa et al, 2024 [20]	Portugal	To assess the feasibility and acceptability of the “Connected We St@nd” online self-management intervention (combining education and psychosocial support) for adults on in-center hemodialysis and family caregivers	Nationwide advertisements on social media, newspapers, mailing lists of support associations	Mixed methods research	Convenience sampling	16	49 (14.2)	Quantitative: questionnaires at baseline and postintervention (PANAS-SF^a^, HADS^b^, PiL-R^c^, WHOQ-BREF^d^, ESRD-AQ^e^) Qualitative: focus group discussion
Young et al, 2024 [21]	United Kingdom	To assess the feasibility and acceptability of the Kidney BEAM^f^ DHI, and explore participants’ perceptions of remote trial procedures and the intervention	Five NHS^g^ hospitals within the UK	Mixed methods research	Quantitative: randomized 1:1 using randomly permuted blocks Qualitative: maximum variation sampling	42	52 (14)	Quantitative: PROMs^h^ (KDQoL-SF1.3^i^, EQ-5D-5L^j^, PAM-13^k^, etc), STS60^l^ test Qualitative: semistructured interviews
Reston et al, 2023 [22]	United Kingdom	To assess the feasibility of a multichannel digital and telephone support program (CareKnowDo) integrated with a patient-facing electronic health record (Patient View), and to inform a fully powered RCT^m^	2 NHS kidney clinics (Gloucestershire Royal Hospital and North Bristol NHS Trust)	Mixed methods research	Purposive sampling	61	56.5 (15.7)	Quantitative: questionnaires at baseline and 6 months (clinical measures, BMQ^n^, B-IPQ^o^, PHQ-9)^p^ Qualitative: semistructured interviews
Pieroni et al, 2023 [23]	Canada	To implement a home telemonitoring system VIEWER^q^ and assess adherence and acceptability of telemonitoring in advanced CKD^r^ patients, and explore patient experiences	Kidney Health Clinic at Seven Oaks General Hospital	Mixed methods research	Purposive sampling	36	57 (12)	Quantitative: daily self-assessment completion rates, satisfaction survey Qualitative: semistructured interviews, focus group discussions
Cheng et al, 2023 [24]	Taiwan, China	To apply an AI LINE Chatbot to improve self-care ability of peritoneal dialysis patients, and evaluate user satisfaction and infection rate changes	Peritoneal Dialysis Center, National Taiwan University Hospital	Quantitative research	Purposive sampling	297	53.6 (13.9)	The questionnaire designed based on the success model of Delone and McLean IS^s^
Zwi et al, 2022 [25]	Australia	To investigate feasibility of the SUCCESS app; a cross-platform e-health innovation to improve health literacy, self-management, and shared decision-making among culturally diverse Australian hemodialysis patients.	Hemodialysis centers in 5 hospitals across 4 local health districts of New South Wales	Mixed methods research	Purposive sampling	61	57.2 (14.6)	Quantitative: health literacy questionnaire, decision self-efficacy scale, KDQOL^t^, health behavior questionnaire, knowledge questionnaire, confidence measure, MAUQ^u^, MoCA^v^, adapted MACL^w^; Qualitative: semistructured interviews
Donald et al, 2022 [26]	Canada	To evaluate participant engagement, perceived self-efficacy, and website usage of a preliminary evaluation of My Kidneys My Health, a patient-facing eHealth tool in Canada.	National kidney disease organizations (Can-SOLVE CKD Network, Kidney Health Strategic Clinical Network, Kidney Foundation of Canada) and outpatient CKD clinics in Alberta	Mixed methods research	Convenience sampling	22	60.6	Quantitative: eHEALS^x^, TAM^y^, CDSES^z^, Google Analytics; Qualitative: semistructured telephone interviews
Shen et al, 2022 [27]	China	To examine perceptions, attitudes, and needs of Chinese patients with CKD and HCPs^aa^ towards eHealth-based self-management interventions in general and the Dutch MD^ab^ intervention specifically	Department of Nephrology, the First Affiliated Hospital of Zhengzhou University	Qualitative study	Snowball sampling	11	Interview mean 38.9 (9.6), focus group mean 43.3 (13.2)	Semistructured interviews focus group discussion
Marinho et al, 2022 [28]	Brazil	To assess smartphone use in patients with CKD on dialysis and their willingness to use mobile applications as a disease self-management strategy	The hemodialysis unit of a renal treatment reference center in the São Francisco Valley region in northeastern Brazil	Quantitative research	Purposive sampling	381	50.8 (16.0)	Self-developed questionnaire
Schrauben et al, 2021 [29]	United States	To assess technology use, attitudes toward using DHT^ac^, and the proficiency in using digital health technologies among individuals with CKD	University of Pennsylvania, Johns Hopkins University, Case Western Reserve University, University of Michigan, University of Illinois at Chicago, Tulane University, and Kaiser Permanente of Northern California	Mixed methods research	Purposive sampling	932	67.9 (9.3)	eHEALS mHealth or technology^ad^ use survey
Toni et al, 2021 [30]	Iran	To understand users’ needs and requirements in CKD care to consider in the design of an ePHR^ae^ to facilitate its implementation, adoption, and use.	Academic hospital of UUMS^af^	Qualitative study	Purposive sampling	15	50.73	Semistructured interviews focus group
Bonner et al, 2018 [31]	Australia	To evaluate current use and type of engagement with DHT (internet and mobile phones), perceived barriers, and opportunities to support CKD self-management.	Five renal services (2 regional, 3 metropolitan) in Queensland, Australia	Quantitative research	Purposive sampling	708	58.3	38-item self-report questionnaire

a PANAS-SF: Positive Affect and Negative Affect Scale Short-Form.

b HADS: Hospital Anxiety and Depression Scale.

c PiL-R: Purpose in Life Test-Revised.

d WHOQ-BREF: World Health Organization's Quality of Life Instruments.

e BREF; ESRD-AQ: End-Stage Renal Disease-Adherence Questionnaire.

f BEAM DHI: BEAM digital health intervention.

g NHS: National Health Service.

h PROMs: Patient Reported Outcome Measures

i KDQoL‑SF1.3: Kidney Disease Quality of Life Short Form 1.3

j EQ‑5D‑5L: EuroQol Five Dimensions Questionnaire with 5 Levels.

k PAM‑13: Patient Activation Measure-13.

l STS60: Sit-to-Stand in Sixty Seconds.

m RCT: randomized controlled trial.

n BMQ: Beliefs about Medicines Questionnaire.

o B-IPQ: Brief Illness Perceptions Questionnaire.

p PHQ-9: Patient Health Questionnaire-9

q VIEWER: virtual ward incorporating electronic wearables.

r CKD: Chronic Kidney Disease.

s IS: information system.

t KDQOL: Kidney Disease Quality of Life.

u MAUQ: mHealth App Usability Questionnaire.

v MoCA: Montreal Cognitive Assessment.

w MACL: Multicomponent Assessment of Computer Literacy.

x eHEALS: eHealth Literacy Scale.

y TAM: Technology Acceptance Model.

z CDSES: Chronic Disease Self-efficacy Scale.

aa HCP: health care professionals.

ab MD: medical dashboard.

ac DHT: Digital Health Technology.

ad mHealth: mobile health.

ae ePHR: electronic personal health record.

af UUMS: Urmia University of Medical Sciences.

### Quality Assessment of the Eligible Studies

Of the included qualitative studies, both were rated as high quality, having met all applicable MMAT criteria. Among the quantitative studies, one cross-sectional study [31] was similarly rated as high quality, while the remaining two quantitative studies (one cross-sectional [28] and one prospective interventional [24]) had methodological limitations due to flaws in measurement tools, failure to account for confounding factors, or a lack of robust sample representativeness. Of the eight mixed methods studies, six [19-21,25,26,29] were rated as high quality using the same criteria, whereas the remaining two [22,23] did not adequately explain the divergence between the qualitative and quantitative findings. Further details are presented in Table 3.

**Table 3.** Quality assessment of the eligible studies (n=13).

Study type							
Qualitative study	S1^a^	S2^b^	1.1^c^	1.2^d^	1.3^e^	1.4^f^	1.5^g^
Toni et al, 2021 [30]	Yes	Yes	Yes	Yes	Yes	Yes	Yes
Shen et al, 2022 [27]	Yes	Yes	Yes	Yes	Yes	Yes	Yes
Prospective interventional study	S1	S2	3.1^h^	3.2^i^	3.3^j^	3.4^k^	3.5^l^
Cheng et al, 2023 [24]	Yes	Yes	Yes	No	Yes	No	Yes
Cross-sectional study	S1	S2	4.1^m^	4.2^n^	4.3^o^	4.4^p^	4.5^q^
Bonner et al, 2018 [31]	Yes	Yes	Yes	Yes	Yes	Yes	Yes
Marinho et al, 2022 [28]	Yes	Yes	Yes	No	Yes	Yes	Yes
Mixed methods research	S1	S2	5.1^r^	5.2^s^	5.3^t^	5.4^u^	5.5^v^
Schrauben et al, 2021 [29]	Yes	Yes	Yes	Yes	Yes	Yes	Yes
Zwi et al, 2022 [25]	Yes	Yes	Yes	Yes	Yes	Yes	Yes
Donald et al, 2022 [26]	Yes	Yes	Yes	Yes	Yes	Yes	Yes
Pieroni et al, 2023 [23]	Yes	Yes	Yes	Yes	No	Unclear	Yes
Reston et al, 2023 [22]	Yes	Yes	Yes	Yes	No	Unclear	Yes
Young et al, 2024 [21]	Yes	Yes	Yes	Yes	Yes	Yes	Yes
Sousa et al, 2024 [20]	Yes	Yes	Yes	Yes	Yes	Yes	Yes
Lightfoot et al, 2025 [19]	Yes	Yes	Yes	Yes	Yes	Yes	Yes

a S1 = Is there a straightforward research question?

b S2 = Whether the collected data can answer the research question. If either S1 or S2 was rated as "no" or "unclear," the article did not need further evaluation.

c 1.1 = Whether qualitative methods were appropriate to answer the research question.

d 1.2 = Whether qualitative data collection methods were adequate to answer the research question.

e 1.3 = Whether the data collected are sufficient to distill the findings of the study.

f 1.4 = Whether the interpretation of the results is supported by sufficient data.

g 1.5 = Whether there is consistency between the source, collection, analysis, and interpretation of qualitative data.

h 3.1 Whether the study subjects are representative of the target population.

i 3.2 Whether the measurements of outcomes and interventions (or exposures) are appropriate.

j 3.3 Whether complete outcome data are available.

k 3.4 Whether confounding factors were considered in the study design and analysis.

l 3.5 Whether the intervention was delivered as intended (or the exposure occurred as expected) during the study period.

m 4.1 = Whether the sampling method was appropriate for answering the research question.

n 4.2 = Whether the sample is representative of the target population.

o 4.3 = Whether the measurement method is appropriate.

p 4.4 = Whether the risk of nonresponse bias is low.

q 4.5 = Whether the statistical analysis method is appropriate.

r 5.1 = Whether there is sufficient justification for using a mixed methods design to address the research question.

s 5.2 = Whether the different components of the study were effectively integrated to answer the research question.

t 5.3 = Whether the results of both the qualitative and quantitative components are adequately explained.

u 5.4 = Whether the issue of disagreement and heterogeneity between quantitative and qualitative results is adequately addressed.

v 5.5 = Whether the different components of the study met the quality criteria involved in each previous routine approach.

### Facilitators and Barriers to DHT-Assisted Self-Management in Patients With CKD

**Figure 2.**
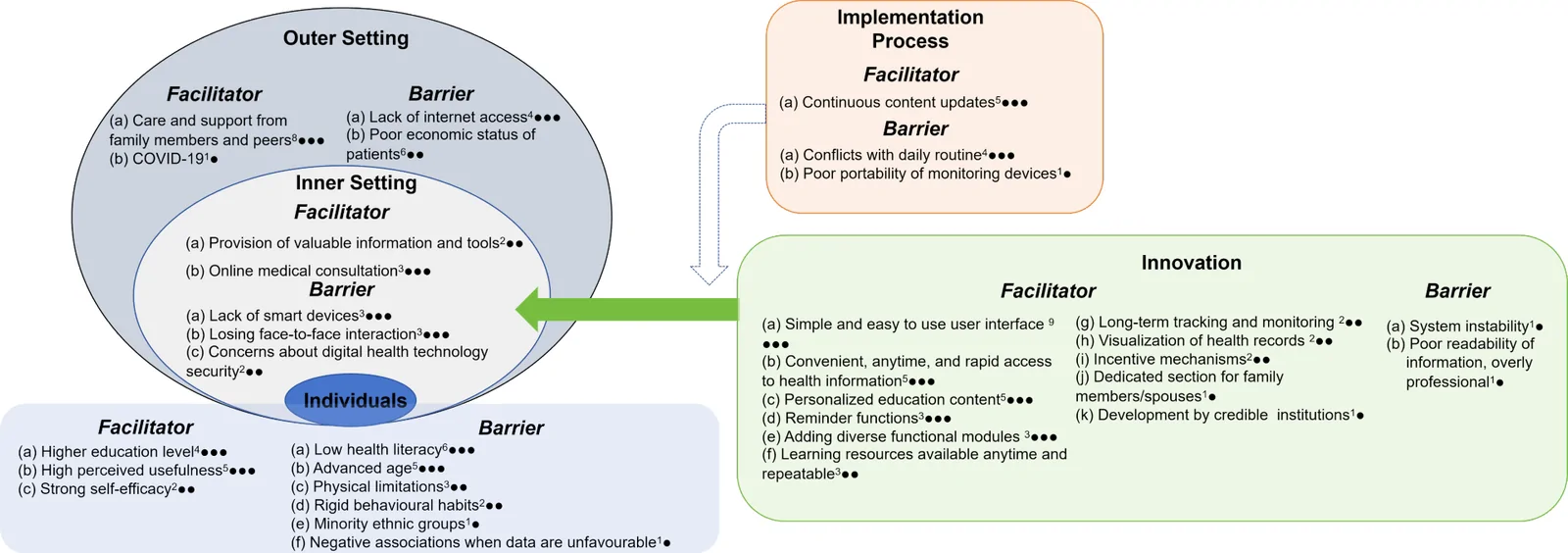
Overview of barriers and facilitators. Numbers represent the number of studies contributing to each factor. Confidence ratings: •••, high; ••, moderate; •, low (based on GRADE-CERQual assessment). GRADE-CERQual: Grading of Recommendations Assessment, Development and Evaluations-Confidence in Evidence from Reviews of Qualitative Research.

In total, 19 facilitators and 15 barriers were identified and mapped to the 5 CFIR 2.0 domains. Among the most frequently reported facilitators were a simple and easy-to-use user interface (9 studies, high confidence), convenient anytime access to health information (5 studies, high confidence), and personalized educational content (5 studies, high confidence) within the innovation domain; care and support from family members and peers (8 studies, high confidence) within the outer setting domain; high perceived usefulness (5 studies, high confidence) within the individual characteristics domain; and continuous content updates (5 studies, high confidence) within the implementation process domain. The most frequently reported barriers were low health literacy (6 studies, high confidence) and advanced age (5 studies, high confidence) within the individual characteristics domain, as well as poor economic status of patients (6 studies, moderate confidence) within the outer setting domain. An overview of the data synthesis, supported by illustrative quotes, is presented in Multimedia Appendix 3. Figure 2 provides a visual summary of the 34 synthesized themes (19 facilitators and 15 barriers), organized by CFIR domain, with the number of supporting studies and confidence ratings for each finding.

## Discussion

### Overview

Based on the CFIR 2.0, this study systematically identified the facilitators and barriers of DHT-assisted self-management in patients with CKD. To the best of our knowledge, the application of the updated CFIR 2.0 framework to synthesize facilitators and barriers of digital health interventions for CKD self-management has not been previously reported. Through the synthesis and analysis of 13 studies involving 3002 patients, a total of 19 facilitators and 15 barriers were identified. These findings indicate that the implementation effectiveness of DHT for self-management in patients with CKD is influenced by multiple factors.

### Innovation Domain

The most consistently reported facilitator was a simple and easy-to-use user interface (9 studies, high confidence) [19-27]. For patients with CKD, who are predominantly older adults with multiple comorbidities, age-related declines in vision, dexterity, and cognitive function can directly affect their ability to interact with digital tools. This is particularly critical given that perceived ease of use is a strong determinant of technology acceptance; once users encounter operational difficulties, their self-efficacy and motivation decline rapidly [32]. An app with a simple and intuitive interface can therefore significantly lower the entry barrier for this population, who often lack experience with complex software.

Convenient, anytime access to health information was also frequently reported (5 studies, high confidence) [19,20,22,27,30]. For patients with CKD, the ability to obtain reliable information on demand is an important enabler of day-to-day self-management. Personalized educational content was similarly identified as a key facilitator (5 studies, high confidence) [19,21,22,26,30], reflecting the need to tailor information to patients’ CKD stage, comorbidities, and health literacy levels.

Reminder functions (3 studies, high confidence) [19,23,30], adding diverse functional modules (3 studies, moderate confidence) [20,25,26], and learning resources available anytime and repeatable (3 studies, moderate confidence) [24-26] together point to a broader theme: the importance of flexibility and adaptability in digital health tools for CKD. Early warning and reminder functions that are easy to implement have been shown to be of significant value for self-management in older adults with chronic conditions [32]. In CKD, where the treatment trajectory spans many years and involves complex medication and dietary regimens, such tailored reminders may be especially impactful in supporting sustained adherence.

Key barriers included poor readability of information and system instability [22], while development by credible institutions, such as governments or hospitals, was identified as a facilitator. A mixed methods study among Australian patients with CKD revealed that over 70% of Australians wish to interact with trustworthy DHT; however, nearly half of the health information delivered is not supported by evidence, the deficiency that directly affects patients’ motivation to use DHT for self-management [33]. For patients with CKD, who navigate an unpredictable disease trajectory, heavy treatment burdens, and years of ongoing self-management, the need is not merely for accessible information, but for trustworthy information that is integrated into their long-term relationship with their care team.

### Outer Setting

Family and peer support was one of the most frequently reported facilitators (8 studies, high confidence) [19-21,23,25,26,29,30]. In CKD, the physical and cognitive demands of dialysis and advanced disease management often necessitate substantial caregiver involvement, making family support not just a facilitator but often a prerequisite for sustained engagement with digital health tools. Peer support offers a distinct advantage rooted in shared lived experience, building trust and enabling patients to exchange practical strategies more closely aligned with daily life [34].

Lack of internet access (4 studies, high confidence) [23,29-31] and poor economic status (6 studies, moderate confidence) [19,23,24,27-29] were reported as barriers. Although these digital health interventions can improve access to care, they may inadvertently widen existing health inequities. This is particularly concerning in CKD, where the disease disproportionately affects socioeconomically disadvantaged populations who may already face limited digital access and financial constraints. Systemic efforts are needed to improve digital health accessibility, including expanding network availability, subsidizing data plans for low-income patients, and integrating digital health support into routine CKD care [35].

COVID-19 was identified as a facilitator in one study (low confidence) [24], reflecting how the pandemic accelerated the adoption of remote communication and patient education. This finding aligns with broader evidence that the pandemic significantly increased digital health usage in CKD care [36,37]. However, given that this is a single low-confidence finding, it should be interpreted with caution and may reflect a context-specific response rather than a sustained shift in practice.

### Inner Setting

Within the inner setting domain, provision of valuable information and tools (2 studies, moderate confidence) [25,29] and online medical consultation (3 studies, high confidence) [26,27,30] were identified as facilitators. Patients valued practical, actionable information and the ability to communicate with their care team through digital platforms. Conversely, loss of face-to-face interaction (3 studies, high confidence) [22,29,31] was reported as a barrier. Studies suggest that patients generally favor a combination of telemedicine and in-person care, particularly for complex or urgent issues [38,39], indicating that digital tools should extend rather than replace the patient-provider relationship.

Lack of smart devices (3 studies, high confidence) [28-30] was identified as a barrier. A cross-sectional survey of 949 patients from 21 hemodialysis centers in the United States found that although 81% owned a smartphone or other internet-enabled device, 19% still lacked access to such devices [40]. These findings suggest that addressing device shortages alone does not translate into meaningful use of digital health resources; broader efforts are needed to ensure equitable access and digital literacy support.

Concerns about digital health security (2 studies, moderate confidence) [29,30] were also reported. Patients expressed worries about constant monitoring and the confidentiality of their health information. These concerns may be particularly pronounced among older adults, who may already be hesitant to adopt new technologies. Transparent data protection policies and clear communication about how patient data are used and secured are therefore essential to foster trust and encourage sustained engagement with digital health tools.

### Individual Characteristics

Patient-level characteristics play a critical role in digital health adoption among individuals with CKD. Higher education level (4 studies, high confidence) [28-31], high perceived usefulness (5 studies, high confidence) [20,24,26,28,30], and strong self-efficacy (2 studies, moderate confidence) [23,30] were identified as facilitators, whereas low health literacy (6 studies, high confidence) [19,21,27,29-31] was the most frequently reported barrier. Low health literacy is especially consequential in CKD, where self-management demands complex knowledge of fluid and dietary restrictions, medication regimens, and symptom monitoring. Patients with lower health literacy tend to prioritize basic functions (eg, disease knowledge and symptom tracking), whereas those with higher literacy prefer more advanced features [41]. This highlights the need for nurses to assess patients’ health literacy before recommending digital tools and to tailor training and support accordingly.

Advanced age (5 studies, high confidence) [24,28-31] compounds these challenges. Older adults often experience psychological distress when adopting new technologies—fear of mistakes, frustration with device operation, or lack of interpersonal interaction—which does not dissipate with increased knowledge alone [42]. Without psychological support and guidance, even highly educated older adults may hesitate to engage with digital health tools. Training programs targeting computer and internet skills can help bridge this gap [43].

Physical limitations (3 studies, moderate confidence) [22,24,29] and rigid behavioral habits (2 studies, moderate confidence) [25,29] were also identified as barriers. Minority ethnic groups (1 study, low confidence) [29] and negative associations when data are unfavorable (1 study, low confidence) [23] were reported in single studies, suggesting that these factors may be context-specific and require further investigation.

### Implementation Process

Within the implementation process domain, continuous content updates (5 studies, high confidence) [19,21,25-27] were identified as the only facilitator, as participants valued timely, relevant information and expressed a desire for alerts when new content became available.

Conflicts with daily routine (4 studies, high confidence) [19-21,25] were a major barrier. The lives of patients with CKD, particularly those on hemodialysis, are highly structured by treatment schedules. Frequent and prolonged dialysis sessions, compounded by fatigue, dietary management, and medication routines, leave little flexibility for additional tasks. If a DHT requires frequent operation, lengthy sessions, or delivers reminders at inappropriate times (eg, during dialysis), it is likely to be perceived as a burden rather than a support. This temporal constraint is relatively unique to dialysis-dependent patients, distinguishing CKD from many other chronic conditions with more flexible treatment schedules, and underscores the need for tools designed to accommodate fixed, demanding routines.

Poor portability of monitoring devices (1 study, low confidence) [23] was also identified as a barrier. Bulky equipment, complex connections, or cumbersome synchronization steps may discourage use outside the home. Streamlining device design could enhance adherence, particularly for patients who travel or have active lifestyles.

We selected CFIR 2.0 for its comprehensive, multilevel structure, which captures determinants across the innovation, individual, organizational, and system levels—extending beyond the individual focus of frameworks such as unified theory of acceptance and use of technology (UTAUT) or Technology Acceptance Model (TAM). Notably, several CFIR constructs—particularly those within the Implementation Process domain (eg, Teaming, Planning, Engaging, Reflecting & Evaluating)—remained unpopulated in our synthesis. This pattern reflects a gap in the current evidence base: existing studies have focused predominantly on patient- and innovation-level factors, with limited attention to organizational and process-oriented determinants. Rather than indicating a limitation of CFIR 2.0, this finding highlights a critical direction for future research.

### Conclusions

In summary, the factors influencing the implementation of digital health interventions among patients with CKD are multidimensional and complex. Guided by the CFIR 2.0 framework, this systematic review synthesized evidence from 13 studies involving 3,002 patients and identified 19 facilitators and 15 barriers across the 5 CFIR domains. Based on these findings and GRADE-CERQual confidence assessments, we offer the following prioritized recommendations, graded as firm (high confidence), conditional (moderate confidence), or suggestive (low confidence). For innovation, developers should ensure simple, intuitive interfaces with clear navigation and plain language (firm), provide personalized education and proactive reminders (firm), ensure anytime, anywhere access to information (firm), and avoid overly technical terminology (suggestive). For outer setting, device and internet access should be addressed through subsidies and WiFi support (firm), and family and peers should be involved as co-users (firm). For inner setting, close patient–provider communication should be maintained via digital platforms alongside face-to-face care (firm), with timely staff reminders (firm), adopt transparent data policies and clear privacy assurances (conditional), and provide subsidized or loaner devices to eligible patients (conditional). For individual characteristics, health and digital literacy should be assessed before tool recommendation, with tailored training (firm); age-adapted support (firm) and adaptive features for physical limitations (conditional) should also be offered. For implementation process, digital tools should align with patients’ daily routines, avoiding demands during dialysis (firm), monitoring devices should be portable (suggestive), and continuous content updates should be maintained (firm). These strategies should be implemented with attention to local context and patient diversity, with future research needed to evaluate their long-term sustainability and real-world effectiveness.

### Limitations and Future Directions

This study has several limitations. First, restricting the search to studies published in English or Chinese may have excluded relevant evidence reported in other languages, introducing potential language bias. Second, although the MMAT guidelines do not recommend excluding studies based on quality scores, the methodological limitations of some included studies may affect the robustness of our conclusions. Third, most studies were conducted in high- or upper-middle-income countries, with limited representation from low-income settings; differences in health care systems, policies, and digital infrastructure may limit the generalizability of our findings. Fourth, the relatively small number of included studies (n=13) limits the strength and generalizability of our findings. Although the total sample across studies was substantial (3002 patients), the predominance of mixed methods and qualitative designs (10 of 13 studies) means that while the evidence offers rich contextual insights, it provides limited quantitative precision for estimating the consistency or magnitude of these factors across different settings and populations.

These limitations point to several directions for future research. First, future systematic reviews should include multilingual searches to reduce language bias and capture a broader range of evidence. Second, more rigorous mixed methods or longitudinal studies are needed. Third, future research should prioritize the development and evaluation of DHT in low-income countries and among underserved populations to enhance the generalizability and equity of findings. Fourth, qualitative and mixed methods studies should incorporate implementation frameworks such as CFIR 2.0 at the design stage to ensure that organizational and process-level determinants are systematically captured, addressing the gaps identified in the current literature.

## Supplementary material

10.2196/103560 Multimedia Appendix 1 Search strategy.

10.2196/103560 Multimedia Appendix 2 Summary of findings and GRADE-CERQual assessments.

10.2196/103560 Multimedia Appendix 3 Additional table with details.

10.2196/103560 Multimedia Appendix 4 List of excluded full-text articles with reasons for exclusion.

10.2196/103560 Multimedia Appendix 5 The complete coding matrix.

10.2196/103560 Checklist 1 PRISMA 2020 checklist.

## Abbreviations

CFIR: Consolidated Framework for Implementation Research
CKD: chronic kidney disease
DHT: Digital health technology
GRADE-CERQual: Grading of Recommendations Assessment, Development and Evaluations-Confidence in Evidence from Reviews of Qualitative research
JBI: Joanna Briggs Institute
KDQOL: kidney disease quality of life
MMAT: Mixed Methods Appraisal Tool
MMSR: Mixed methods systematic review
PICOS: population, interest of phenomenon, context, and study design
PRESS: peer review of electronic search strategies
PRISMA: Preferred reporting items for systematic reviews and meta-analysis
PROMs: patient reported outcome measures
RCT: randomized controlled trial
TAM: Technology acceptance model
UTAUT: Unified theory of acceptance and use of technology

## References

[1] Zhu W, Han M, Wang Y, Wang G (2024). Trend analysis and prediction of the incidence and mortality of CKD in China and the US. BMC Nephrol.

[2] Ortiz A, Lees JS, Torra R, Stel VS, Kramer A, Mark PB (2026). The updated global burden of chronic kidney disease: one death every 20 seconds. Nephrol Dial Transplant.

[3] Van de Velde D, De Zutter F, Satink T (2019). Delineating the concept of self-management in chronic conditions: a concept analysis. BMJ Open.

[4] Mengjiao L, Xujie Z, Ping J (2025). Self-management assessment tools for people with hypertension: a scoping review. BMC Nephrol.

[5] Baay S, Hemmelgarn B, Tam-Tham H (2019). Understanding adults with chronic kidney disease and their caregivers’ self-management experiences: a qualitative study using the theoretical domains framework. Can J Kidney Health Dis.

[6] Vase L, Wager TD, Eccleston C (2025). Opportunities for chronic pain self-management: core psychological principles and neurobiological underpinnings. Lancet.

[7] Hwang M, Zheng Y, Cho Y, Jiang Y (2025). AI applications for chronic condition self-management: scoping review. J Med Internet Res.

[8] Shen H, van der Kleij R, van der Boog PJM, Chang X, Chavannes NH (2019). Electronic health self-management interventions for patients with chronic kidney disease:systematic review of quantitative and qualitative evidence. J Med Internet Res.

[9] Badr J, Motulsky A, Denis JL (2024). Digital health technologies and inequalities: a scoping review of potential impacts and policy recommendations. Health Policy.

[10] Campbell J, Porter J (2015). Dietary mobile apps and their effect on nutritional indicators in chronic renal disease: a systematic review. Nephrology (Carlton).

[11] Paneerselvam GS, Lua PL, Chooi WH, Rehman IU, Goh KW, Ming LC (2025). Effectiveness of mobile apps in improving medication adherence among chronic kidney disease patients: systematic review. J Med Internet Res.

[12] Zhu J, Xie J, Luo Y (2025). The impact of digital health interventions on psychological health, self-efficacy, and quality of life in patients with end-stage kidney disease: systematic review and meta-analysis. J Med Internet Res.

[13] Damschroder LJ, Reardon CM, Widerquist MAO, Lowery J (2022). The updated consolidated framework for implementation research based on user feedback. Implement Sci.

[14] Page MJ, McKenzie JE, Bossuyt PM (2021). The PRISMA 2020 statement: an updated guideline for reporting systematic reviews. BMJ.

[15] Stern C, Lizarondo L, Carrier J (2021). Methodological guidance for the conduct of mixed methods systematic reviews. JBI Evid Implement.

[16] Jorgenson A, Adalberto Luz R, Fábrega Juskevicius L, Clara Padoveze M, Price L (2022). The consolidated framework for implementation research: a reflection on researchers’ experiences of its benefits and challenges and the lessons learnt from using it. Nurse Res.

[17] McGowan J, Sampson M, Salzwedel DM, Cogo E, Foerster V, Lefebvre C (2016). PRESS peer review of electronic search strategies: 2015 guideline statement. J Clin Epidemiol.

[18] Hong QN, Gonzalez-Reyes A, Pluye P (2018). Improving the usefulness of a tool for appraising the quality of qualitative, quantitative and mixed methods studies, the Mixed Methods Appraisal Tool (MMAT). J Eval Clin Pract.

[19] Lightfoot CJ, Wilkinson TJ, Billany RE (2025). Use, usability, and experience testing of a digital health intervention to support chronic kidney disease self-management: mixed methods study. J Med Internet Res.

[20] Sousa H, Ribeiro O, Bártolo A (2024). The connected We St@nd programme: a feasibility pilot study of an online self-management intervention for adults on in-centre haemodialysis and family caregivers. Br J Health Psychol.

[21] Young HML, Castle EM, Briggs J (2024). The development and internal pilot trial of a digital physical activity and emotional well-being intervention (Kidney BEAM) for people with chronic kidney disease. Sci Rep.

[22] Reston RE, Caskey FJ, Hole B, Udayaraj U, Weinman J (2023). CareKnowDo-a multichannel digital and telephone support program for people with chronic kidney disease: feasibility randomized controlled trial. JMIR Form Res.

[23] Pieroni D, Leon SJ, Krueger AL (2023). Use of wearable and wireless technology in real-world clinical settings to improve patient outcomes in chronic kidney disease: a mixed methods pilot prospective trial. Can J Kidney Health Dis.

[24] Cheng CI, Lin WJ, Liu HT, Chen YT, Chiang CK, Hung KY (2023). Implementation of artificial intelligence chatbot in peritoneal dialysis nursing care: experience from a Taiwan medical center. Nephrology (Carlton).

[25] Zwi S, Isautier J, Webster AC (2022). A feasibility study of a best practice health literacy app for Australian adults with chronic kidney disease. PEC Innov.

[26] Donald M, Beanlands H, Straus S (2022). An eHealth self-management intervention for adults with chronic kidney disease, My Kidneys My Health: a mixed-methods study. CMAJ Open.

[27] Shen H, van der Kleij R, van der Boog PJM (2022). Digital tools/eHealth to support CKD self-management: a qualitative study of perceptions, attitudes and needs of patients and health care professionals in China. Int J Med Inform.

[28] Marinho CLA, Gomes OV, Silva Junior G da, Schwingel PA (2022). Smartphone and application use in self-management of chronic kidney disease: a cross-sectional feasibility study. Sao Paulo Med J.

[29] Schrauben SJ, Appel L, Rivera E (2021). Mobile Health (mHealth) technology: assessment of availability, acceptability, and use in CKD. Am J Kidney Dis.

[30] Toni E, Pirnejad H, Makhdoomi K, Mivefroshan A, Niazkhani Z (2021). Patient empowerment through a user-centered design of an electronic personal health record: a qualitative study of user requirements in chronic kidney disease. BMC Med Inform Decis Mak.

[31] Bonner A, Gillespie K, Campbell KL (2018). Evaluating the prevalence and opportunity for technology use in chronic kidney disease patients: a cross-sectional study. BMC Nephrol.

[32] Graeber J, Warmerdam E, Aufenberg S (2026). Technology acceptance of 2 mHealth apps during an observational technology evaluation study: qualitative results of a mixed methods study. JMIR Hum Factors.

[33] Catapan S de C, Taylor ML, Scuffham P, Smith AC, Kelly JT (2025). Improving consumer trust in digital health: a mixed methods study involving people living with chronic kidney disease. Digit Health.

[34] Kasan N, Amriati Y, Chanif C (2025). Family support interventions in hemodialysis patients:a systematic review. JN.

[35] Saifan AR, Sawalha M, Alarabyat IA (2026). Beyond access: telehealth readiness, trust, and early use among Jordanian patients with chronic illness. Health Care (Don Mills).

[36] Nouri S, Lyles CR, Sherwin EB (2023). Visit and between-visit interaction frequency before and after COVID-19 telehealth implementation. JAMA Netw Open.

[37] Santosh R, Mohammed YN, Rahaman Z, Khurana S (2024). The role of telemedicine in enhancing Chronic Kidney Disease (CKD) management and dialysis care. Cureus.

[38] Moulaei K, Sheikhtaheri A, Fatehi F, Shanbehzadeh M, Bahaadinbeigy K (2023). Patients’ perspectives and preferences toward telemedicine versus in-person visits: a mixed-methods study on 1226 patients. BMC Med Inform Decis Mak.

[39] Danila MI, Gavigan K, Rivera E (2022). Patient perceptions and preferences regarding telemedicine for autoimmune rheumatic diseases care during the COVID ‐19 pandemic. Arthritis Care Res (Hoboken).

[40] Hussein WF, Bennett PN, Pace S (2020). The mobile health readiness of people receiving in-center hemodialysis and home dialysis. Clin J Am Soc Nephrol.

[41] Gao Y, Pu LJ, Yan Y (2026). Demand analysis of mobile health applications for patients with chronic kidney disease across different e-health literacy Llevels. Patient Prefer Adherence.

[42] Hepburn J, Williams L, McCann L (2025). Barriers to and facilitators of digital health technology adoption among older adults with chronic diseases: updated systematic review. JMIR Aging.

[43] Wu S, Wan Z, Peng L, Zhang Q, Wu X, Lin X (2026). Awareness, want, and adoption of digital health services among older adults in China: a cross-sectional study. Front Public Health.

