# Supplementary material for: Facilitators and Barriers to Digital Health Technologies for Self-Management in Patients With Chronic Kidney Disease: Systematic Review Based on the Updated CFIR 2.0 Framework
Source: J Med Internet Res. 2026 Sep 21;28:e103560. doi: 10.2196/103560 (PMC13592748; doi:10.2196/103560)
Supplement: Supplementary file 1 [file jmir-v28-e103560-s001.docx]

| Search strategy | | | |
| --- | --- | --- | --- |
| Database | **Date** | **Search strategy** | **Hits** |
| PUBMED |  |  |  |
|  | 12-05-2026 | ( ("Renal Insufficiency, Chronic"[Mesh] OR "Kidney Failure, Chronic"[Mesh] OR "Renal Dialysis"[Mesh] OR "chronic kidney disease"[Title/Abstract] OR CKD[Title/Abstract] OR "renal insufficiency"[Title/Abstract] OR "kidney disease"[Title/Abstract] OR dialysis[Title/Abstract] OR hemodialysis[Title/Abstract] OR haemodialysis[Title/Abstract] OR "end stage renal disease"[Title/Abstract] OR ESRD[Title/Abstract])) AND ( ("Telemedicine"[Mesh] OR "Mobile Applications"[Mesh] OR "Smartphone"[Mesh] OR "Computers, Handheld"[Mesh] OR "Internet"[Mesh] OR mHealth[Title/Abstract] OR "mobile health"[Title/Abstract] OR "eHealth"[Title/Abstract] OR telehealth[Title/Abstract] OR "mobile app*"[Title/Abstract] OR "smartphone"[Title/Abstract] OR "health app*"[Title/Abstract] OR "web-based"[Title/Abstract] OR internet[Title/Abstract] OR digital[Title/Abstract] OR "digital health"[Title/Abstract])) AND ( ("Self-Management"[Mesh] OR "Self Care"[Mesh] OR self-manag*[Title/Abstract] OR self-care[Title/Abstract] OR "self monitor*"[Title/Abstract] OR "self efficacy"[Title/Abstract])) AND ( ( "Qualitative Research"[Mesh] OR "Cross-Sectional Studies"[Mesh] OR "Randomized Controlled Trial"[Mesh] OR "Controlled Clinical Trial"[Mesh] OR "qualitative"[Title/Abstract] OR "cross-sectional"[Title/Abstract] OR "cross sectional"[Title/Abstract] OR "mixed method"[Title/Abstract] OR "mixed-methods"[Title/Abstract] OR "multimethod"[Title/Abstract] OR "quantitative and qualitative"[Title/Abstract] OR "qualitative and quantitative"[Title/Abstract] OR "randomized controlled trial"[Title/Abstract] OR "RCT"[Title/Abstract] OR "prospective"[Title/Abstract] OR "cohort"[Title/Abstract] OR "longitudinal"[Title/Abstract] OR "barrier"[Title/Abstract] OR "facilitator"[Title/Abstract] OR "challenge*"[Title/Abstract] OR "enabler"[Title/Abstract] OR "experience"[Title/Abstract] OR "perception"[Title/Abstract] OR "perspective"[Title/Abstract] OR "attitude"[Title/Abstract] OR "view"[Title/Abstract] OR "obstacle"[Title/Abstract] OR "encourage"[Title/Abstract] OR "hinder"[Title/Abstract] OR "discourage"[Title/Abstract])) | 158 |
| EMBASE |  |  |  |
|  | 12-05-2026 | ('chronic kidney failure'/exp OR 'dialysis'/exp OR 'chronic kidney disease':ab,ti,kw OR ckd:ab,ti,kw OR 'renal insufficiency':ab,ti,kw OR 'kidney disease':ab,ti,kw OR hemodialysis:ab,ti,kw OR haemodialysis:ab,ti,kw OR 'end stage renal':ab,ti,kw OR esrd:ab,ti,kw) AND ('telemedicine'/exp OR 'mobile application'/exp OR 'smartphone'/exp OR 'internet'/exp OR mhealth:ab,ti,kw OR 'mobile health':ab,ti,kw OR ehealth:ab,ti,kw OR telehealth:ab,ti,kw OR 'mobile app*':ab,ti,kw OR smartphone:ab,ti,kw OR 'health app*':ab,ti,kw OR 'web-based':ab,ti,kw OR internet:ab,ti,kw OR digital:ab,ti,kw OR 'digital health':ab,ti,kw) AND ('self care'/exp OR 'self-management':ab,ti,kw OR self-manag*:ab,ti,kw OR 'self-care':ab,ti,kw OR 'self monitor*':ab,ti,kw OR 'self-efficacy':ab,ti,kw) AND ('qualitative research'/exp OR 'cross-sectional study'/exp OR 'randomized controlled trial'/exp OR 'qualitative':ab,ti,kw OR 'cross-sectional':ab,ti,kw OR 'cross sectional':ab,ti,kw OR 'mixed method':ab,ti,kw OR 'mixed-methods':ab,ti,kw OR 'multimethod':ab,ti,kw OR 'quantitative and qualitative':ab,ti,kw OR 'qualitative and quantitative':ab,ti,kw OR 'randomized controlled trial':ab,ti,kw OR 'rct':ab,ti,kw OR 'prospective':ab,ti,kw OR 'cohort':ab,ti,kw OR 'longitudinal':ab,ti,kw OR 'barrier*':ab,ti,kw OR 'facilitator*':ab,ti,kw OR 'challenge*':ab,ti,kw OR 'enabler*':ab,ti,kw OR 'experience*':ab,ti,kw OR 'perception*':ab,ti,kw OR 'perspective*':ab,ti,kw OR 'attitude*':ab,ti,kw OR 'view*':ab,ti,kw OR 'obstacle':ab,ti,kw OR 'encourage':ab,ti,kw OR 'hinder':ab,ti,kw OR 'discourage':ab,ti,kw) | 328 |
| CINAHL |  |  |  |
|  | 12-05-2026 | #1 (MH “Renal Dialysis+”) OR (MH “Chronic Kidney Disease+”) OR (MH “End Stage Renal Disease”) OR TI ( (“chronic kidney” OR CKD OR dialysis OR hemodialysis OR haemodialysis OR ESRD) ) OR AB ( (“chronic kidney” OR CKD OR dialysis OR hemodialysis OR haemodialysis OR ESRD) ) (41,997)  #2 (MH “Telehealth”) OR (MH “Mobile Applications”) OR (MH “Computers, Handheld”) OR (MH “Internet”) OR TI ( (mHealth OR “mobile health” OR eHealth OR telehealth OR “mobile app*” OR smartphone OR “digital health”) ) OR AB ( (mHealth OR “mobile health” OR eHealth OR telehealth OR “mobile app*” OR smartphone OR “digital health”) ) (91,653)  #3 (MH “Self-Management+”) OR (MH “Self Care+”) OR (MH “Self Efficacy”) OR TI ( (self-manag* OR self-care OR “self monitor*” OR self-efficacy) ) OR AB ( (self-manag* OR self-care OR “self monitor*” OR self-efficacy) ) (89,857)  #4 (MH "Qualitative Studies+") OR (MH "Cross Sectional Studies") OR (MH "Randomized Controlled Trials") OR TI ("qualitative" OR "cross-sectional" OR "cross sectional" OR "mixed method" OR "mixed-methods" OR "multimethod" OR "quantitative and qualitative" OR "qualitative and quantitative" OR "randomized controlled trial" OR "RCT" OR "prospective" OR "cohort" OR "longitudinal" OR "barrier*" OR "facilitator*" OR "challenge*" OR "enabler*" OR "experience*" OR "perception*" OR "perspective*" OR "attitude*" OR "view*" OR "obstacle" OR "encourage" OR "hinder" OR "discourage") OR AB ("qualitative" OR "cross-sectional" OR "cross sectional" OR "mixed method" OR "mixed-methods" OR "multimethod" OR "quantitative and qualitative" OR "qualitative and quantitative" OR "randomized controlled trial" OR "RCT" OR "prospective" OR "cohort" OR "longitudinal" OR "barrier*" OR "facilitator*" OR "challenge*" OR "enabler*" OR "experience*" OR "perception*" OR "perspective*" OR "attitude*" OR "view*" OR "obstacle" OR "encourage" OR "hinder" OR "discourage") (1,742,263)  #5 :#1 AND #2 AND #3 AND #4 | 35 |
| Cochrane | | |  |
|  | 12-05-2026 | #1 MeSH descriptor: [Renal Insufficiency, Chronic] this term only (10,538)  #2 MeSH descriptor: [Renal Dialysis] this term only (7,667)  #3 (chronic kidney disease or CKD or renal insufficiency or kidney disease or dialysis or hemodialysis or haemodialysis or end stage renal or ESRD):ti,ab,kw (60,270)  #4: #1 OR #2 OR #3 (60,935)  #5 MeSH descriptor: [Telemedicine] this term only (6,490)  #6 MeSH descriptor: [Mobile Applications] this term only (3,129)  #7 MeSH descriptor: [Smartphone] this term only (1,482)  #8 MeSH descriptor: [Internet] this term only (1,482)  #9 (mHealth or mobile health or eHealth or telehealth or telemedicine or mobile app* or smartphone or health app* or web-based or internet or digital or digital health):ti,ab,kw (7,354)  #10 :#5 OR #6 OR #7 OR #8 OR #9 (216,866)  #11 MeSH descriptor: [Self-Management] this term only (1,679)  #12 MeSH descriptor: [Self Care] this term only (8,234)  #13 (self-manag* or self-care or self monitor* or self efficacy):ti,ab,kw (84,625)  #14 :#11 OR #12 OR #13 (85,403)  #15 MeSH descriptor: [Qualitative Research] this term only (3,144)  #16 MeSH descriptor: [Cross-Sectional Studies] this term only (10,447)  #17 MeSH descriptor: [Randomized Controlled Trial] this term only (34)  #18 MeSH descriptor: [Controlled Clinical Trial] this term only (37)  #19 (qualitative OR cross-sectional OR cross sectional OR mixed method OR mixed-methods OR multimethod OR quantitative and qualitative OR qualitative and quantitative OR randomized controlled trial OR RCT OR prospective OR cohort OR longitudinal OR barrier* OR facilitator* OR challenge* OR enabler* OR experience* OR perception* OR perspective* OR attitude* OR view* OR obstacle OR encourage OR hinder OR discourage):ti,ab,kw (1238,601)  #20 :#15 OR #16 OR #17 OR #18 OR #19 (1238,604)  #21 :#4 AND #10 AND #14 AND #20 (340) | 340 |
| Web of Science | | |  |
|  | 12-05-2026 | TS=(Qualitative Research OR Cross-Sectional Studies OR Randomized Controlled Trial OR Controlled Clinical Trial OR qualitative OR cross-sectional OR cross sectional OR mixed method OR mixed-methods OR multimethod OR quantitative and qualitative OR qualitative and quantitative OR randomized controlled trial OR RCT OR prospective OR cohort OR longitudinal OR barrier* OR facilitator* OR challenge* OR enabler* OR experience* OR perception* OR perspective* OR attitude* OR view* OR obstacle OR encourage OR hinder OR discourage OR Health Knowledge) AND TS=(Self-Management OR Self Care OR self-manag* OR self-care OR self monitor* OR self efficacy) AND TS=(Telemedicine OR Mobile Applications OR Smartphone OR Computers, Handheld OR Internet OR mHealth OR mobile health OR eHealth OR telehealth OR mobile app* OR smartphone OR health app* OR web-based OR internet OR digital OR digital health) AND TS=(Renal Insufficiency, Chronic OR Kidney Failure, Chronic OR Renal Dialysis OR chronic kidney disease OR CKD OR renal insufficiency OR kidney disease OR dialysis OR hemodialysis OR haemodialysis OR end stage renal disease OR ESRD) | 131 |
| PsycINFO | | | 2 |
|  | c | #1 (MH “Renal Dialysis+”) OR (MH “Chronic Kidney Disease+”) OR (MH “End Stage Renal Disease”) OR TI ( (“chronic kidney” OR CKD OR dialysis OR hemodialysis OR haemodialysis OR ESRD) ) OR AB ( (“chronic kidney” OR CKD OR dialysis OR hemodialysis OR haemodialysis OR ESRD) ) (4265)  #2 (MH “Telehealth”) OR (MH “Mobile Applications”) OR (MH “Computers, Handheld”) OR (MH “Internet”) OR TI ( (mHealth OR “mobile health” OR eHealth OR telehealth OR “mobile app*” OR smartphone OR “digital health”) ) OR AB ( (mHealth OR “mobile health” OR eHealth OR telehealth OR “mobile app*” OR smartphone OR “digital health”) ) (15,005)  #3 (MH “Self-Management+”) OR (MH “Self Care+”) OR (MH “Self Efficacy”) OR TI ( (self-manag* OR self-care OR “self monitor*” OR self-efficacy) ) OR AB ( (self-manag* OR self-care OR “self monitor*” OR self-efficacy) ) (78,293)  #4 (MH "Qualitative Studies+") OR (MH "Cross Sectional Studies") OR (MH "Randomized Controlled Trials") OR TI ("qualitative" OR "cross-sectional" OR "cross sectional" OR "mixed method" OR "mixed-methods" OR "multimethod" OR "quantitative and qualitative" OR "qualitative and quantitative" OR "randomized controlled trial" OR "RCT" OR "prospective" OR "cohort" OR "longitudinal" OR "barrier*" OR "facilitator*" OR "challenge*" OR "enabler*" OR "experience*" OR "perception*" OR "perspective*" OR "attitude*" OR "view*" OR "obstacle" OR "encourage" OR "hinder" OR "discourage") OR AB ("qualitative" OR "cross-sectional" OR "cross sectional" OR "mixed method" OR "mixed-methods" OR "multimethod" OR "quantitative and qualitative" OR "qualitative and quantitative" OR "randomized controlled trial" OR "RCT" OR "prospective" OR "cohort" OR "longitudinal" OR "barrier*" OR "facilitator*" OR "challenge*" OR "enabler*" OR "experience*" OR "perception*" OR "perspective*" OR "attitude*" OR "view*" OR "obstacle" OR "encourage" OR "hinder" OR "discourage")) (1,773,512)  #5 :#1 AND #2 AND #3 AND #4 |  |
| Scopus | | | |
|  | 11-05-2026 | #1 TITLE-ABS-KEY("Renal Insufficiency, Chronic" OR "Kidney Failure, Chronic" OR "Renal Dialysis" OR "chronic kidney disease" OR CKD OR "renal insufficiency" OR "kidney disease" OR dialysis OR hemodialysis OR haemodialysis OR "end stage renal disease" OR ESRD) (577,372)  #2 TITLE-ABS-KEY("Telemedicine" OR "Mobile Applications" OR "Smartphone" OR "Computers, Handheld" OR "Internet" OR mHealth OR "mobile health" OR eHealth OR telehealth OR "mobile app*" OR smartphone OR "health app*" OR "web-based" OR internet OR digital OR "digital health") (2,387,775)  #3 TITLE-ABS-KEY("Self-Management" OR "Self Care" OR "self-manag*" OR "self-care" OR "self monitor*" OR "self efficacy") (174,497)  #4 TITLE-ABS-KEY("Qualitative Research" OR "Cross-Sectional Studies" OR "Randomized Controlled Trial" OR qualitative OR cross-sectional OR "cross sectional" OR "mixed method" OR "mixed-methods" OR multimethod OR "quantitative and qualitative" OR "qualitative and quantitative" OR "randomized controlled trial" OR RCT OR prospective OR cohort OR longitudinal OR barrier* OR facilitator* OR challenge* OR enabler* OR experience* OR perception* OR perspective* OR attitude* OR view* OR obstacle OR encourage OR hinder OR discourage) (10,154,524)  #5:#1 AND #2 AND #3 AND #4 | 90 |
| CNKI | | | |
|  |  | SU=('慢性肾病' + '慢性肾脏病' + '慢性肾功能不全' + '肾功能衰竭' + '终末期肾病' + '终末期肾脏病' + '尿毒症' + '透析' + '血液透析' + '腹膜透析' + '肾透析' + 'CKD' + 'ESRD' + '慢性肾衰' + '肾衰竭') * ('移动健康' + '远程医疗' + '互联网医疗' + '移动医疗' + '智能手机' + '移动应用' + '手机应用' + '应用程序' + 'APP' + '健康APP' + '数字化健康' + '数字健康' + '互联网' +'在线' + '网络' + '远程' + '居家监测' ＋ '小程序' + 'mHealth' + 'telehealth' + 'eHealth') * ('自我管理' + '自我照护' + '自我护理' + '自我照顾' + '自我效能' + '自我监测' + '自我监督' + '疾病管理' + '健康管理') * ('质性研究' + '定性研究' + '访谈' + '横断面' + '现况调查' + '混合方法' + '随机对照' + 'RCT' + '前瞻性' + '队列' + '纵向' + '促进因素' + '阻碍因素' + '障碍因素' + '影响因素' + '相关因素' + '体验'+ '认知' + '态度' + '需求' + '满意度') | 172 |
| Wanfang | | | |
|  |  | 主题:("慢性肾病" or "慢性肾脏病" or "慢性肾功能不全" or "肾功能衰竭" or "终末期肾病" or "终末期肾脏病" or "尿毒症" or "透析" or "血液透析" or "腹膜透析" or "肾透析" or "CKD" or "ESRD" or "慢性肾衰" or "肾衰竭") and 主题:("移动健康" or "远程医疗" or "互联网医疗" or "移动医疗" or "智能手机" or "移动应用" or "手机应用" or "应用程序" or "APP" or "健康APP" or "数字化健康" or "数字健康" or "互联网" or "在线" or "网络" or "远程" or "居家监测" or "小程序" or "mHealth" or "telehealth" or "eHealth") and 主题:("自我管理" or "自我照护" or "自我护理" or "自我照顾" or "自我效能" or "自我监测" or "自我监督" or "疾病管理" or "健康管理") and 主题:("质性研究" or "定性研究" or "访谈" or "横断面" or "现况调查" or "混合方法" or "随机对照" or "RCT" or "前瞻性" or "队列" or "纵向" or "促进因素" or "阻碍因素" or "障碍因素" or "影响因素" or "相关因素" or "体验" or "认知" or "态度" or "需求" or "满意度") | 49 |
| VIP | | | |
|  |  | M=(移动健康 OR m健康 OR 移动医疗 OR 智能手机 OR 健康APP OR 移动应用 OR 互联网) AND M=(慢性肾病 OR 透析 OR 肾病 OR 终末期肾病 OR 肾功能) AND M=(自我管理 OR 自我照护 OR 自我护理 OR 健康管理) | 56 |
| Chinese Medical Journal database | | | |
|  |  | #1 全部字段=("慢性肾病" OR "慢性肾脏病" OR "慢性肾功能不全" OR "肾功能衰竭" OR "终末期肾病" OR "终末期肾脏病" OR "尿毒症" OR "透析" OR "血液透析" OR "腹膜透析" OR "肾透析" OR "CKD" OR "ESRD" OR "慢性肾衰" OR "肾衰竭") 20155  #2全部字段="移动健康" OR "远程医疗" OR "互联网医疗" OR "移动医疗" OR "智能手机" OR "移动应用" OR "手机应用" OR "应用程序" OR "APP" OR "健康APP" OR "数字化健康" OR "数字健康" OR "互联网" OR "在线" OR "网络" OR "远程" OR "居家监测" OR "小程序" OR "mHealth" OR "telehealth" OR "eHealth" 18947  #3全部字段=("自我管理" OR "自我照护" OR "自我护理" OR "自我照顾" OR "自我效能" OR "自我监测" OR "自我监督" OR "疾病管理" OR "健康管理") 10577  #4全部字段=("质性研究" OR "定性研究" OR "访谈" OR "横断面" OR "现况调查" OR "混合方法" OR "随机对照" OR "RCT" OR "前瞻性" OR "队列" OR "纵向" OR "促进因素" OR "阻碍因素" OR "障碍因素" OR "影响因素" OR "相关因素" OR "体验" OR "认知" OR "态度" OR "需求" OR "满意度") (131,685)  #5:#1 AND #2 AND #3 AND #4 | 4 |
