# Supplementary material for: Facilitators and Barriers to Digital Health Technologies for Self-Management in Patients With Chronic Kidney Disease: Systematic Review Based on the Updated CFIR 2.0 Framework
Source: J Med Internet Res. 2026 Sep 21;28:e103560. doi: 10.2196/103560 (PMC13592748; doi:10.2196/103560)
Supplement: Supplementary file 2 [file jmir-v28-e103560-s002.docx]

**Summary of findings and GRADE-CERQual assessments**

| **Summary of findings** | **Studies contributing to the findings** | **Assessment** | | | | **Overall CERQual assessment** | | **Explanation of CERQual assessment** |
| --- | --- | --- | --- | --- | --- | --- | --- | --- |
|  |  | **Methodological limitations** | **Relevance** | **Coherence** | **Adequacy** |  |  |  |
| *CFIR construct 1. Innovation source* | | | | | | | | |
| Development by credible institutions | (Shen et al., 2022) | No or very minor concerns (single high-quality qualitative study; MMAT 1.1–1.5 all Yes) | No or very minor concerns (directly relevant to the theme) | No or very minor concerns (findings were contextually or conceptually coherent) | Moderate-to-serious concerns(a single study) | Low confidence | | This finding was graded as low confidence because of moderate-to-serious concerns regarding adequacy. |
| *CFIR construct 2. Innovation relative advantage* | | | | | | | | |
| Convenient, anytime, and rapid access to health information | (Lightfoot et al., 2025; Sousa et al., 2024; Shen et al., 2022; Toni et al., 2021; Reston et al., 2023) | Minor concerns (one study with moderate concerns [Reston et al., 2023: MMAT 5.3 No, 5.4 Unclear]; 4 studies with no concerns) | No or very minor concerns (all studies directly relevant to the theme) | No or very minor concerns (findings were contextually or conceptually coherent) | Minor concerns (5 studies offered moderately rich data overall) | High confidence | | This finding was graded as high confidence because only minor concerns were identified. |
| *CFIR construct 3. Innovation adaptability* | | | | | | | | |
| Personalized education content | (Lightfoot et al., 2025; Young et al., 2024; Reston et al., 2023; Toni et al., 2021; Donald et al., 2022) | Minor concerns(one study with moderate concerns [Reston et al., 2023: MMAT 5.3 No; 5.4 Unclear]; 4 studies with no concerns) | No or very minor concerns (all studies directly relevant to the theme) | No or very minor concerns (findings were contextually or conceptually coherent) | Minor concerns (5 studies offered moderately rich data overall) | High confidence | | This finding was graded as high confidence because only minor concerns were identified. |
| Poor readability of information, overly professional | Reston et al., 2023 | Moderate concerns (single study with moderate methodological concerns [Reston et al., 2023: MMAT 5.3 No; 5.4 Unclear] | No or very minor concerns (directly relevant to theme) | No or very minor concerns (findings were contextually or conceptually coherent) | Moderate-to-serious concerns(a single study) | Low confidence | | This finding was graded as low confidence because of moderate-to-serious concerns regarding adequacy. |
| *CFIR construct 4.Innovation complexity* | | | | | | | | |
| Simple and easy to use user interface | (Lightfoot et al., 2025; Young et al., 2024; Reston et al., 2023; Sousa et al., 2024; Cheng et al., 2023; Zwi et al., 2022; Donald et al., 2022; Shen et al., 2022; Pieroni et al., 2023) | Minor concerns (two studies with moderate concerns [Reston et al., 2023: MMAT 5.3 No; 5.4 Unclear; Pieroni et al., 2023: 5.3 No; 5.4 Unclear]; one study with serious concerns [Cheng et al., 2023: 3.2 No; 3.4 No]; 6 studies with no methodological concerns) | No or very minor concerns (directly relevant to theme) | No or very minor concerns (findings were contextually or conceptually coherent) | No or very minor concerns (9 studies offered rich data overall) | High confidence | | This finding was graded as high confidence because only minor concerns were identified. |
| System instability | Reston et al., 2023 | Moderate concerns (single study with moderate methodological concerns [Reston et al., 2023: MMAT 5.3 No; 5.4 Unclear] | No or very minor concerns (directly relevant to theme) | No or very minor concerns (findings were contextually or conceptually coherent) | Moderate-to-serious concerns(a single study) | Low confidence | | This finding was graded as low confidence because of moderate-to-serious concerns regarding adequacy. |
| *CFIR construct 5. Innovation design* | | | | | | | | |
| Reminder functions | (Toni et al., 2021; Pieroni et al., 2023; Lightfoot et al., 2025) | Minor concerns (one study with moderate concerns [Pieroni et al., 2023: MMAT 5.3 No; 5.4 Unclear]; 2 studies with no methodological concerns) | No or very minor concerns (directly relevant to theme) | No or very minor concerns (findings were contextually or conceptually coherent) | Minor concerns (3 studies offered moderately rich data overall) | High confidence | | This finding was graded as high confidence because only minor concerns were identified. |
| Adding diverse functional modules | (Donald et al., 2022; Zwi et al., 2022; Sousa et al., 2024) | No or very minor concerns (3 studies with no methodological concerns) | No or very minor concerns (directly relevant to theme) | No or very minor concerns (findings were contextually or conceptually coherent) | Minor concerns (3 studies offered moderately rich data overall) | High confidence | | This finding was graded as high confidence because only minor concerns were identified. |
| Learning resources available anytime and repeatable | (Donald et al., 2022; Zwi et al., 2022; Cheng et al., 2023) | Moderate concerns (one study with serious concerns [Cheng et al., 2023: MMAT 3.2 No; 3.4 No]; 2studies with no methodological concerns.) | No or very minor concerns (directly relevant to theme) | No or very minor concerns (findings were contextually or conceptually coherent) | Minor concerns (3 studies offered moderately rich data overall) | Moderrate confidence | | This finding was graded as moderate confidence because of moderate concerns regarding methodological limitations. |
| Long‑term tracking and monitoring | (Shen et al., 2022; Young et al., 2024) | No or very minor concerns (two studies with no methodological concerns) | No or very minor concerns (directly relevant to theme) | No or very minor concerns (findings were contextually or conceptually coherent) | Moderate concerns (2 studies; limited data richness on this specific theme) | Moderate confidence | | This finding was graded as moderate confidence because of moderate concerns regarding adequacy. |
| Visualization of health records | (Pieroni et al., 2023; Lightfoot et al., 2025) | Moderate concerns (one study with moderate concerns [Pieroni et al., 2023: 5.3 No; 5.4 Unclear]; one study with no methodological concerns) | No or very minor concerns (directly relevant to theme) | No or very minor concerns (findings were contextually or conceptually coherent) | Moderate concerns (2 studies; limited data richness on this specific theme) | Moderate confidence | | This finding was graded as moderate confidence because of moderate concerns regarding adequacy and methodological limitations. |
| Incentive mechanisms | (Young et al., 2024; Lightfoot et al., 2025) | No or very minor concerns (two studies with no methodological concerns) | No or very minor concerns (directly relevant to theme) | No or very minor concerns (findings were contextually or conceptually coherent) | Moderate concerns (2 studies; limited data richness on this specific theme) | Moderate confidence | | This finding was graded as moderate confidence because of moderate concerns regarding adequacy. |
| Dedicated section for family members/spouses | Donald et al., 2022 | No or very minor concerns (single high-quality mixed-methods study; MMAT 5.1–5.5 all Yes) | No or very minor concerns (directly relevant to theme) | No or very minor concerns (findings were contextually or conceptually coherent) | Moderate-to-serious concerns(a single study) | Low confidence | | This finding was graded as low confidence because of moderate-to-serious concerns regarding adequacy. |
| *CFIR construct 6. Critical incidents* | | | | | | | | |
| COVID-19 | Cheng et al., 2023 | Moderate-to-serious concerns (MMAT 3.2 No; 3.4 No]) | No or very minor concerns (directly relevant to theme) | No or very minor concerns (findings were contextually or conceptually coherent) | Moderate-to-serious concerns(a single study) | Low confidence | | This finding was graded as low confidence because of moderate-to-serious concerns regarding adequacy and methodological limitations. |
| *CFIR construct 7. Local conditions* | | | | | | | | |
| Lack of internet access | (Bonner et al., 2018; Toni et al., 2021; Schrauben et al., 2021; Pieroni et al., 2023) | Minor concerns (one study with moderate concerns [Pieroni et al., 2023: MMAT 5.3 No; 5.4 Unclear]; 3 studies with no methodological concerns) | No or very minor concerns (directly relevant to theme) | No or very minor concerns (findings were contextually or conceptually coherent) | Minor concerns (4 studies offered moderately rich data overall) | High confidence | | This finding was graded as high confidence because only minor concerns were identified. |
| *CFIR construct 8. Partnerships & connections* | | | | | | | | |
| Care and support from family members and peers | (Toni et al., 2021; Schrauben et al., 2021; Donald et al., 2022; Zwi et al., 2022; Pieroni et al., 2023; Young et al., 2024; Sousa et al., 2024; Lightfoot et al., 2025) | Minor concerns (one study with moderate concerns [Pieroni et al., 2023: MMAT 5.3 No; 5.4 Unclear]; 7 studies with no methodological concerns) | No or very minor concerns (directly relevant to theme) | No or very minor concerns (findings were contextually or conceptually coherent) | No or very minor concerns (8 studies offered rich data overall) | High confidence | | This finding was graded as high confidence because only minor concerns were identified. |
| *CFIR construct 9. Financing* | | | | | | | | |
| Poor economic status of patients | (Schrauben et al., 2021; Marinho et al., 2022; Shen et al., 2022; Cheng et al., 2023; Pieroni et al., 2023; Lightfoot et al., 2025) | Moderate concerns ( one study with moderate concerns [Marinho et al., 2022: MMAT 4.2 No]; one study with moderate concerns [Pieroni et al., 2023: MMAT 5.3 No; 5.4 Unclear]; one study with serious concerns [Cheng et al., 2023: MMAT 3.2 No; 3.4 No]; 3 studies with no methodological concerns.) | No or very minor concerns (directly relevant to theme) | No or very minor concerns (findings were contextually or conceptually coherent) | Minor concerns (6 studies offered moderately rich data overall) | Moderate confidence | This finding was graded as moderate confidence because of moderate concerns regarding methodological limitations. | |
| *CFIR construct 10. Compatibility* | | | | | | | | |
| Concerns about DHT security | (Schrauben et al., 2021; Toni et al., 2021) | No or very minor concerns (2 studies with no methodological concerns) | No or very minor concerns (directly relevant to theme) | No or very minor concerns (findings were contextually or conceptually coherent) | Moderate concerns (2 studies; limited data richness on this specific theme) | Moderate confidence | This finding was graded as moderate confidence because of moderate concerns regarding adequacy. | |
| *CFIR construct 11. Relational connections* | | | | | | | | |
| Online medical consultatio | (Toni et al., 2021; Shen et al., 2022; Donald et al., 2022) | No or very minor concerns (3 studies with no methodological concerns) | No or very minor concerns (directly relevant to theme) | No or very minor concerns (findings were contextually or conceptually coherent) | Minor concerns (3 studies offered moderately rich data overall) | High confidence | This finding was graded as high confidence because only minor concerns were identified. | |
| *CFIR construct 12. Communications* | | | | | | | | |
| Losing face-to-face interaction | (Bonner et al., 2018; Schrauben et al., 2021; Reston et al., 2023) | Minor concerns (one study with moderate concerns [Reston et al., 2023: MMAT 5.3 No; 5.4 Unclear]; 2 studies with no methodological concerns) | No or very minor concerns (directly relevant to theme) | No or very minor concerns (findings were contextually or conceptually coherent) | Minor concerns (3 studies offered moderately rich data overall) | High confidence | This finding was graded as high confidence because only minor concerns were identified. | |
| *CFIR construct 13. Access to knowledge & information* | | | | | | | | |
| Provision of valuable information and tools | (Schrauben et al., 2021; Zwi et al., 2022) | No or very minor concerns (2 studies with no methodological concerns) | No or very minor concerns (directly relevant to theme) | No or very minor concerns (findings were contextually or conceptually coherent) | Moderate concerns (2 studies; limited data richness on this specific theme) | Moderate confidence | This finding was graded as moderate confidence because of moderate concerns regarding adequacy. | |
| *CFIR construct 14. Available resources (Materials & equipment)* | | | | | | | | |
| Lack of smart devices | (Toni et al., 2021; Schrauben et al., 2021; Marinho et al., 2022) | Minor concerns (one study with moderate concerns [Marinho et al., 2022: MMAT 4.2 No]; 2 studies with no methodological concerns) | No or very minor concerns (directly relevant to theme) | No or very minor concerns (findings were contextually or conceptually coherent) | Minor concerns (3 studies offered moderately rich data overall) | High confidence | This finding was graded as high confidence because only minor concerns were identified. | |
| *CFIR construct 15. Individuals (Innovation Recipients)* | | | | | | | | |
| Higher education level | (Bonner et al., 2018; Toni et al., 2021; Schrauben et al., 2021; Marinho et al., 2022) | Minor concerns (one study with moderate concerns [Marinho et al., 2022: 4.2 No]; 3 studies with no methodological concerns) | No or very minor concerns (directly relevant to theme) | No or very minor concerns (findings were contextually or conceptually coherent) | Minor concerns (4 studies offered moderately rich data overall) | High confidence | This finding was graded as high confidence because only minor concerns were identified. | |
| Advanced age | (Bonner et al., 2018; Toni et al., 2021; Schrauben et al., 2021; Marinho et al., 2022; Cheng et al., 2023) | Minor concerns (one study with moderate concerns [Marinho et al., 2022: MMAT 4.2 No]; one study with serious concerns [Cheng et al., 2023: MMAT 3.2 No; 3.4 No]; 3 studies with no methodological concerns; ) | No or very minor concerns (directly relevant to theme) | No or very minor concerns (findings were contextually or conceptually coherent) | Minor concerns (5 studies offered moderately rich data overall) | High confidence | This finding was graded as high confidence because only minor concerns were identified. | |
| Minority ethnic groups | (Schrauben et al., 2021) | No or very minor concerns (single high-quality mixed-methods study; MMAT 5.1–5.5 all Yes) | No or very minor concerns (directly relevant to theme) | No or very minor concerns (findings were contextually or conceptually coherent) | Moderate-to-serious concerns(a single study) | Low confidence | This finding was graded as low confidence because of moderate-to-serious concerns regarding adequacy. | |
| *CFIR construct 16. Characteristics (Need)* | | | | | | | | |
| Strong self- efficacy | (Toni et al., 2021; Pieroni et al., 2023) | Moderate concerns (one study with moderate concerns [Pieroni et al., 2023: MMAT 5.3 No; 5.4 Unclear]; one study with no methodological concerns) | No or very minor concerns (directly relevant to theme) | No or very minor concerns (findings were contextually or conceptually coherent) | Moderate concerns (2 studies; limited data richness on this specific theme) | Moderate confidence | This finding was graded as moderate confidence because of moderate concerns regarding adequacy and methodological limitations. | |
| *CFIR construct 17. Characteristics (Capability)* | | | | | | | | |
| Low health literacy | (Bonner et al., 2018; Toni et al., 2021; Schrauben et al., 2021; Shen et al., 2022; Young et al., 2024; Lightfoot et al., 2025) | No or very minor concerns (6 studies with no methodological concerns) | No or very minor concerns (directly relevant to theme) | No or very minor concerns (findings were contextually or conceptually coherent) | Minor concerns (6 studies offered moderately rich data overall) | High confidence | This finding was graded as high confidence because only minor concerns were identified. | |
| Physical limitations | (Schrauben et al., 2021; Cheng et al., 2023; Reston et al., 2023) | Moderate concerns (one study with moderate concerns [Reston et al., 2023: MMAT 5.3 No; 5.4 Unclear]; one study with serious concerns [Cheng et al., 2023: MMAT 3.2 No; 3.4 No]; one study with no methodological concerns.) | No or very minor concerns (directly relevant to theme) | No or very minor concerns (findings were contextually or conceptually coherent) | Minor concerns (3 studies offered moderately rich data overall) | Moderate confidence | This finding was graded as moderate confidence because of moderate concerns regarding methodological limitations. | |
| Rigid behavioural habits | (Schrauben et al., 2021; Zwi et al., 2022) | No or very minor concerns (2 studies with no methodological concerns) | No or very minor concerns (directly relevant to theme) | No or very minor concerns (findings were contextually or conceptually coherent) | Moderate concerns (2 studies; limited data richness on this specific theme) | Moderate confidence | This finding was graded as moderate confidence because of moderate concerns regarding adequacy. | |
| *CFIR construct 18. Characteristics (Motivation)* | | | | | | | | |
| Negative associations when data are unfavourable | Pieroni et al., 2023 | Moderate concerns (MMAT 5.3 No; 5.4 Unclear) | No or very minor concerns (directly relevant to theme) | No or very minor concerns (findings were contextually or conceptually coherent) | Moderate-to-serious concerns(a single study) | Low confidence | This finding was graded as low confidence because of moderate-to-serious concerns regarding adequacy. | |
| High perceived usefulness | (Toni et al., 2021; Marinho et al., 2022; Donald et al., 2022; Cheng et al., 2023; Sousa et al., 2024) | Minor concerns (one study with moderate concerns [Marinho et al., 2022: MMAT 4.2 No]; one study with serious concerns [Cheng et al., 2023: MMAT 3.2 No; 3.4 No]; 3 studies with no methodological concerns.) | No or very minor concerns (directly relevant to theme) | No or very minor concerns (findings were contextually or conceptually coherent) | Minor concerns (5 studies offered moderately rich data overall) | High confidence | This finding was graded as high confidence because only minor concerns were identified. | |
| *CFIR construct 19. Doing* | | | | | | | | |
| Conflicts with daily routine | (Zwi et al., 2022; Sousa et al., 2024; Young et al., 2024; Lightfoot et al., 2025) | No or very minor concerns (4 studies with no methodological concerns) | No or very minor concerns (directly relevant to theme) | No or very minor concerns (findings were contextually or conceptually coherent) | Minor concerns (4 studies offered moderately rich data overall) | High confidence | This finding was graded as high confidence because only minor concerns were identified. | |
| Poor portability of monitoring devices | (Pieroni et al., 2023) | Moderate concerns (MMAT 5.3 No; 5.4 Unclear) | No or very minor concerns (directly relevant to theme) | No or very minor concerns (findings were contextually or conceptually coherent) | Moderate-to-serious concerns(a single study) | Low confidence | This finding was graded as low confidence because of moderate-to-serious concerns regarding adequacy. | |
| *CFIR construct 20. Adapting* | | | | | | | | |
| Continuous content updates | (Donald et al., 2022; Shen et al., 2022; Zwi et al., 2022; Young et al., 2024; Lightfoot et al., 2025) | No or very minor concerns (5 studies with no methodological concerns) | No or very minor concerns (directly relevant to theme) | No or very minor concerns (findings were contextually or conceptually coherent) | Minor concerns (5 studies offered moderately rich data overall) | High confidence | This finding was graded as high confidence because only minor concerns were identified. | |

Notes: Methodological limitations: For each contributing study, concerns were classified based on MMAT criteria (Table 3): all "Yes" → no concerns; one "Unclear" → minor concerns; one "No" + one "Unclear" → moderate concerns; two or more "No" → serious concerns. For each synthesized finding, the overall rating was determined by the proportion of studies with no concerns: all studies → no or very minor concerns; more than half (> ½) → minor concerns; at least one study, but ≤ half (≤ ½) → moderate concerns; all studies had concerns → the most severe level present was applied. Adequacy: Graded by the number of supporting studies: 1 study → moderate-to-serious concerns; 2 studies → moderate concerns; 3-7 studies → minor concerns; >7 studies → no-to-minor concerns.

REFERENCES:

1. **19.** Lightfoot CJ, Wilkinson TJ, Billany RE, et al. Use, usability, and experience testing of a digital health intervention to support chronic kidney disease self-management: mixed methods study. *J Med Internet Res.* Jun 19, 2025;27:e75845. [doi: ] [Medline: 40537087]
2. **20.** Sousa H, Ribeiro O, Bártolo A, et al. The connected We St@nd programme: a feasibility pilot study of an online self-management intervention for adults on in-centre haemodialysis and family caregivers. *Br J Health Psychol.* Sep 2024;29(3):589-608. [doi: ] [Medline: 38361177]
3. **21.** Young HML, Castle EM, Briggs J, et al. The development and internal pilot trial of a digital physical activity and emotional well-being intervention (Kidney BEAM) for people with chronic kidney disease. *Sci Rep.* Jan 6, 2024;14(1):700. [doi: ] [Medline: 38184737]
4. **22.** Reston RE, Caskey FJ, Hole B, Udayaraj U, Weinman J. CareKnowDo-a multichannel digital and telephone support program for people with chronic kidney disease: feasibility randomized controlled trial. *JMIR Form Res.* Nov 23, 2023;7:e33147. [doi: ] [Medline: 37995117]
5. **23.** Pieroni D, Leon SJ, Krueger AL, et al. Use of wearable and wireless technology in real-world clinical settings to improve patient outcomes in chronic kidney disease: a mixed methods pilot prospective trial. *Can J Kidney Health Dis.* 2023;10:20543581231212125. [doi: ] [Medline: 38020485]
6. **24.** Cheng CI, Lin WJ, Liu HT, Chen YT, Chiang CK, Hung KY. Implementation of artificial intelligence chatbot in peritoneal dialysis nursing care: experience from a Taiwan medical center. *Nephrology (Carlton).* Dec 2023;28(12):655-662. [doi: ] [Medline: 37698229]
7. **25.** Zwi S, Isautier J, Webster AC, et al. A feasibility study of a best practice health literacy app for Australian adults with chronic kidney disease. *PEC Innov.* Dec 2022;1:100047. [doi: ] [Medline: 37213763]
8. **26.** Donald M, Beanlands H, Straus S, et al. An eHealth self-management intervention for adults with chronic kidney disease, My Kidneys My Health: a mixed-methods study. *CMAJ Open.* 2022;10(3):E746-E754. [doi: ] [Medline: 35973709]
9. **27.** Shen H, van der Kleij R, van der Boog PJM, et al. Digital tools/eHealth to support CKD self-management: a qualitative study of perceptions, attitudes and needs of patients and health care professionals in China. *Int J Med Inform.* Sep 2022;165:104811. [doi: ] [Medline: 35753175]
10. **28.** Marinho CLA, Gomes OV, Silva Junior G da, Schwingel PA. Smartphone and application use in self-management of chronic kidney disease: a cross-sectional feasibility study. *Sao Paulo Med J.* 2022;141(4):e202278. [doi: ] [Medline: 36197350]
11. **29.** Schrauben SJ, Appel L, Rivera E, et al. Mobile Health (mHealth) technology: assessment of availability, acceptability, and use in CKD. *Am J Kidney Dis.* Jun 2021;77(6):941-950. [doi: ] [Medline: 33309860]
12. **30.** Toni E, Pirnejad H, Makhdoomi K, Mivefroshan A, Niazkhani Z. Patient empowerment through a user-centered design of an electronic personal health record: a qualitative study of user requirements in chronic kidney disease. *BMC Med Inform Decis Mak.* Nov 24, 2021;21(1):329. [doi: ] [Medline: 34819050]
13. **31.** Bonner A, Gillespie K, Campbell KL, et al. Evaluating the prevalence and opportunity for technology use in chronic kidney disease patients: a cross-sectional study. *BMC Nephrol.* Feb 2, 2018;19(1):28. [doi: ] [Medline: 29394930]
