# Supplementary material for: Facilitators and Barriers to Digital Health Technologies for Self-Management in Patients With Chronic Kidney Disease: Systematic Review Based on the Updated CFIR 2.0 Framework
Source: J Med Internet Res. 2026 Sep 21;28:e103560. doi: 10.2196/103560 (PMC13592748; doi:10.2196/103560)
Supplement: Supplementary file 3 [file jmir-v28-e103560-s003.docx]

Overview of the data synthesis supported by illustrative quotes.

|  | CFIR 2.0 domain, construct, and subconstruct | Barriers (–) and facilitators (+) with illustrative quotes |
| --- | --- | --- |
| **Innovation** | | |
|  | Innovation source | - Development by credible institutions (+) |
|  |  | Specifically, patients mentioned that if interventions were developed by credible eHealth developers such as the government or hospitals, it would facilitate their eHealth use.[27] |
|  | Innovation evidence base | N/A |
|  | Innovation relative advantage | - Convenient, anytime, and rapid access to health information (+) |
|  |  | "Using an ePHR I can quickly access the right information." (A stage 5 patient under hemodialysis).[30] |
|  |  | "It will be convenient if I can see my laboratory tests directly. [...] Especially when doctors are too busy to provide test results." (Patient 15, female, 41y, CKD G4; focus group 2). [27] |
|  |  | "Very useful to have blood test results instantly." ; "Helps you see your results quickly." (patient). [22] |
|  |  | The asynchrony of the educational component was another positive aspect, as it allowed greater flexibility to watch the educational videos and read the booklets at their best convenience.[20] |
|  |  | "You can just, whenever you've got five or ten minutes go on it wherever you are, so I did find that useful. " (Participant SS23; female; 54 y; high activation). [19] |
|  | Innovation adaptability | - Personalized education content (+) |
|  |  | There was an agreement that the information in the ePHR should be displayed according to an individual patient’s condition in a simple and easy to understand language and format. [30] |
|  |  | Tailored tools to actively engage people in executing lifestyle changes and promote enhanced communication with health care providers. [26] |
|  |  | A future iteration of the program should tailor to whether patients are incident or prevalent or to perceived knowledge about the condition. [22] |
|  |  | Provide more CKD stage and life stage specific content. [21] |
|  |  | Being able to tailor and personalize the program to suit their needs was considered an important facilitator. [19] |
|  |  | - Poor readability of information, overly professional (-) |
|  |  | "The website information seems to be written by medical experts and can only be understood by medical experts." (patient) [22] |
|  | Innovation trialability | N/A |
|  | Innovation complexity | - Simple and easy to use user interface (+) |
|  |  | "[Monitoring parameters in] the app is easier and much more convenient than recording them in a notebook." (Patient 7, female, 32y, CKD G5 with peritoneal dialysis; interviews). [27] |
|  |  | Overall, participants commented that MKMH was user friendly and easy to navigate.[26] |
|  |  | Overall, simplicity and ease‑of‑use were features of apps considered to be most important. [25] |
|  |  | 94.9% of patients found the Chatbot easy to use.[24] |
|  |  | "Very easy and comfortable to use! I would highly recommend [VIEWER] to all CKD patients. You don’t have to be computerliterate to use [VIEWER]." [Study participant 0026] [23] |
|  |  | "It was fairly clear, and clear cut. All the information was pretty clearly displayed on there. And it was relatively easy to get to everything." (patient) [22] |
|  |  | This resource was considered easy to navigate, well designed and visually pleasing. [20] |
|  |  | "I like the way it's set out, it's very easy. Everything is within one window and you click on one and it pops up with more information." (Participant SS13; female; 57 y; high activation) [19] |
|  |  | "The overall kind of layout was good, if not a little bit confusing to navigate! " (Female, 20s, White British, haemodialysis, moderate adherence) [21] |
|  |  | - System instability (-) |
|  |  | Follow‑up RN calls revealed that the most common reasons were as follows: not receiving a PV log‑in before attempting to enroll; losing invitation emails in spam folders; not checking emails; and technical difficulties while logging in. [22] |
|  | Innovation design | - Reminder functions (medication, follow‑up visits, vaccination, laboratory results, prolonged inactivity) (+) |
|  |  | They also requested a reminder system due to the chronicity of their disease and frequency of events and preventive measures, for example, to remind the time of medication use, upcoming follow-up visits, vaccinations, laboratory tests checkups, and notification of result availability. [30] |
|  |  | "If it [VIEWER/iPad] had a beep or something to say that now is the time to do on onto this, or this and record it." (FG2 participant) [23] |
|  |  | As part of the MK&M program, participants received automated reminder emails when new content (ie, the ‘How to…’ sessions) was available to them. Participants also received automated reminder emails if they had not logged in after 7, 14, and 28 days.[19] |
|  |  | - Adding diverse functional modules (+) |
|  |  | Suggested changes to the website included more advice and resources regarding travel, mental and sexual health, risk prediction and peer support. [26] |
|  |  | Content suggestions included adding a mental health section. [25] |
|  |  | "This programme helped me to manage my emotions as I was always irritable and nervous. I have always focused on the negative but now I do not see treatment as a sacrifice anymore." [person on haemodialysis]. [20] |
|  |  | - Learning resources available anytime and repeatable (+) |
|  |  | Information is at your fingertips: "You can search for a food and instantly know sodium, phosphorus and potassium." (Patient 16). [26] |
|  |  | For others, however, there was benefit in having an accessible resource to return to multiple times. [25] |
|  |  | I can now access ... anytime, anywhere using my smart device. I no longer have to worry about time or space constraints. [24] |
|  |  | - Long‑term tracking and monitoring (+) |
|  |  | "[Monitoring parameters in] the app is easier and much more convenient than recording them in a notebook." (Patient 7, female, 32y, CKD G5 with peritoneal dialysis; interviews) [27] |
|  |  | "[The diary] was useful because it's quite nice to be able to look back through what you've done. " (Female, 20s, White British, haemodialysis, moderate adherence) [21] |
|  |  | - Visualization of health records (+) |
|  |  | Participants enjoyed the ability to collect vitals and plot trends, which they found helpful in managing their health. [23] |
|  |  | Having graphs to visually display their data was considered helpful. [19] |
|  |  | - Incentive mechanisms (+) |
|  |  | "I like a goal and I'm better with a target!" (Female, 50s, White British, haemodialysis, low adherence). [21] |
|  |  | "The tracker's great because it does incentivise you, definitely, I always want to try." (Participant SS07; male; 55 y; low activation). [19] |
|  |  | - Dedicated section for family members/spouses (+) |
|  |  | Providing webinars and creating content specific to caregivers (i.e., family and friends). [26] |
|  | Innovation cost | N/A |
| **Outer setting** | | |
|  | Critical incidents | - COVID-19 (+) |
|  |  | Due to the COVID-19 pandemic, remote and efficient communication, and patient education are crucial. [24] |
|  | Local attitudes | N/A |
|  | Local conditions | - Lack of internet access (-) |
|  |  | A large number of participants also indicated that hospitals should provide free access to WiFi (n=464,70.3%). [31] |
|  |  | Several patients reported their inability to use or access the technical equipment to use the ePHR (e.g., the Internet or smartphones). [30] |
|  |  | "Do not have internet access" (Female, Age 59, Record 1). [29] |
|  |  | Requires wireless Internet, a resource that may be unavailable in rural and remote communities. [23] |
|  | Partnerships & connections | - Care and support from family members and peers (+) |
|  |  | They, however, believed that having supporters such as caregivers and their family members would assist them to bypass such barriers. [30] |
|  |  | "Would need assistance from spouse for email/internet." (Male, Age 69, Record 68). [29] |
|  |  | "If there’s something interactive on it. … webinar a patient forum so that people can feel safe chatting with other patients." (Patient 18). [26] |
|  |  | Content suggestions included adding a mental health section incorporating peer stories to learn about things that have worked in the past with other patients. [25] |
|  |  | "I was very fortunate because my daughter was with me because she was really supportive of this kit." (FG2 participant). [23] |
|  |  | "My partner watched a few of the informational videos and joined in with a few of the exercises if we got them up on the big telly. " (Female, 20s, White British, haemodialysis, moderate adherence). [21] |
|  |  | "The new experiences and solutions that were exchanged in each session and the support of the psychologists along with the sharing of our [peers] lives and experiences exceeded my expectations in every way." [person on haemodialysis]. [20] |
|  |  | Other participants were receptive to the chat forum and found the peer support received beneficial. [19] |
|  | Policies & laws | N/A |
|  | Financing | - Poor economic status of patients (-) |
|  |  | Younger age (per 10 years) and higher annual household income (≥$20,000 vs. <$20,000) were associated with more current use of technology. [29] |
|  |  | In the present study, this percentage was smaller, which points to the lower purchasing power of patients on dialysis in Brazil. [28] |
|  |  | Some patients would be worried that if they submit abnormal data they would be asked to come to the hospital for a checkup, providing them with a financial burden. [27] |
|  |  | If the software uses more than 500 text messages, it needs to pay 70 US dollars/month. [24] |
|  |  | Several patients noted that they would never have been able to afford devices with similar functionality on their own. Indeed, the cost of commercially available devices was cited as a ‘barrier to entry’ for those interested in monitoring on their own. [23] |
|  |  | Individuals with poor digital literacy and without access to digital technologies, particularly those with lower socioeconomic status and living in rural areas, may face greater challenges in adopting and using DHIs. [19] |
|  | External pressure | N/A |
|  | (1) Societal pressure |  |
|  | (2) Performance-measurement pressure |  |
|  | (3) Market pressure |  |
| **Inner setting** | | |
|  | Structural characteristics | N/A |
|  | (1) Physical infrastructure |  |
|  | (2) Information Technology infrastructure |  |
|  | (3) Work infrastructure |  |
|  | Culture | N/A |
|  | (1) Human equality-centeredness |  |
|  | (2) Recipient-centeredness |  |
|  | (3) Deliverer-centeredness |  |
|  | (4) Learning-centeredness |  |
|  | Compatibility | - Concerns about DHT security (-) |
|  |  | "Do not want any kind of constant monitoring" (Female, Age 61, Record 9). [29] |
|  |  | They recommended establishing mechanisms to strictly define the levels of access to protect privacy and information confidentiality.[30] |
|  | Relative priority | N/A |
|  | Mission alignment | N/A |
|  | Tension for change | N/A |
|  | Relational connections | - Online medical consultation (+) |
|  |  | "If it is possible for me to take a photo from the wound [to share it with my doctor through an ePHR] and my doctor says whether or not I need to come to Urmia, it will be great." (A stage 5 peritoneal dialysis patient). [30] |
|  |  | "Doctors can know our [disease] status at home. We can communicate with doctors directly online." (Patient 20, female, 43y, CKD G2; focus group 1). [27] |
|  |  | One feature that was not apparent to some individuals was the prompt aid for use with health care providers (My Question List) and how to use it. [26] |
|  | Communications | - Losing face-to-face interaction (-) |
|  |  | That is still a preference for face-to-face interaction with health care providers.[31] |
|  |  | "I love coming to the clinic and the interaction between the personal contact with all of the staff" (Female, Age 80, Record 44). [29] |
|  |  | "Better to speak to a person" (patient). [22] |
|  | Access to knowledge & information | - Provision of valuable information and tools (+) |
|  |  | "Good instruction, including video would be important for me to make sure I was doing it correctly" (Male, Age 51, Record 291). [29] |
|  |  | Participants reported most frequently accessing the Health Information section, particularly information on diet and fluids. [25] |
|  | Incentive systems | N/A |
|  | Available resources |  |
|  | (1) Funding | N/A |
|  | (2) Space | N/A |
|  | (3) Materials & equipment | - Lack of smart devices (-) |
|  |  | Several patients reported their inability to use or access the technical equipment to use the ePHR (e.g., the Internet or smartphones). [30] |
|  |  | "Do not own a smartphone but is willing to learn" (Female, Age 66, Record 22). [29] |
|  |  | A total of 381 patients were included, of whom 64% had a smartphone, although only 3.1% knew of a kidney disease-related application. [28] |
| **Individuals** | | |
|  | Individuals |  |
|  | (1) High-level leaders | N/A |
|  | (2) Mid-level leaders | N/A |
|  | (3) Operation leaders | N/A |
|  | (4) Implementation facilitators | N/A |
|  | (5) Implementation leads | N/A |
|  | (6) Implementation team members | N/A |
|  | (7) Other implementation support | N/A |
|  | (8) Innovation deliverers | N/A |
|  | (9) Innovation Recipients | - Higher education level (+) |
|  |  | Those who had obtained higher levels of education had almost double the odds of complex use. [31] |
|  |  | "Many patients are old or illiterate and this will prevent them from accessing the [ePHR] system." (A stage 1 CKD patient) [30] |
|  |  | High school education or higher (vs. less) was associated with more current use of technology. [29] |
|  |  | Only educational attainment remained an independent factor in smartphone. [28] |
|  |  | - Advanced age (-) |
|  |  | Participants aged ≤60 had over six times the odds of using their mobile phone for more complex tasks than did those aged 61 and over. [31] |
|  |  | Yet, many concerned that the advancing age and low e-health literacy would represent the main barriers to adopt the system. [30] |
|  |  | Younger age (per 10 years) was the only other characteristic associated with more interest in future use of digital and mHealth technologies. [29] |
|  |  | The mean age of the patients who had a smartphone (44.7±13.5 years) was statistically lower (*P*<0.001) than that of the patients who did not have one (61.7±14.2 years). [28] |
|  |  | It is not friendly to people with poor eyesight or the elderly. [24] |
|  |  | - Minority ethnic groups (-) |
|  |  | Non-Hispanic Black participants reported less internet/email use than Non-Hispanic white participants. [29] |
|  | Characteristics |  |
|  | (1) Need | - Strong self- efficacy (+) |
|  |  | Our participants had positive attitudes towards the availability of an ePHR. They thought that it can increase their awareness of their disease and the care they needed. [30] |
|  |  | "The best thing was probably you know seeing how many steps I did that day and knowing that I have to take more and watching my weight and trying to keep it within a certain thing." (FG2 participant). [23] |
|  | (2) Capability | - Low health literacy (-) |
|  |  | A total of 217 participants (30.6%) reported that they did not use the internet, of those more than half (n=118,54.4%) reported that they did not know how to use the internet. [31] |
|  |  | Most of their patients have lowere-health literacy and therefore may not be able to understand or use these types of systems. [30] |
|  |  | Only 27% had adequate eHealth literacy (eHEALS score ≥32). [29] |
|  |  | "I always use the smartphone to get a call or read the news. It is difficult if I need to enter websites." (Patient 12, male, 62y, CKD G3; focus group 2). [27] |
|  |  | "Up to 20% of the general population do not possess fundamental digital skills, resources, motivation and confidence needed to effectively use DHIs. [21] |
|  |  | It is necessary to acknowledge that digital poverty exists, with disparities in access to and proficiency in using digital devices and technology. Individuals with poor digital literacy and without access to digital technologies, particularly those with lower socioeconomic status and living in rural areas, may face greater challenges in adopting and using DHIs. [19] |
|  |  | - Physical limitations (e.g., stroke, inability to focus eyes for long periods) (-) |
|  |  | "Had stroke 3 years ago and now have problems trying to read on the internet" (Female, Age 76, Record 326). [29] |
|  |  | It is not friendly to people with poor eyesight or the elderly. [24] |
|  |  | "I haven't felt well enough to stare a computer screen as it brings on the fatigue." (patient). [22] |
|  |  | - Rigid behavioural habits of CKD patients (-) |
|  |  | "I am old school and would rather write." (Male, Age 64, Record 91). [29] |
|  |  | "I'm not going to change my habits now." (55-60 years, male, dialysis vintage > 15 years). [25] |
|  | (3) Opportunity | N/A |
|  | (4) Motivation | - Negative associations when data are unfavourable (-) |
|  |  | Some participants felt discouraged when they observed adverse trends in measurements (eg, rising BP or weight). These negative trends reminded patients that they had a chronic progressive illness. [23] |
|  |  | - High perceived usefulness (+) |
|  |  | "For example, I can be informed about which foods have the highest amount of potassium and how much I can eat in my diet?" (A stage 5 patient under hemodialysis)[30] |
|  |  | Believes that mobile applications may help manage the kidney disease: Yes 226 (59.3%). [28] |
|  |  | Most agreed or strongly agreed that the website contained useful content for dealing with the consequences of CKD.[26] |
|  |  | Patients, and caregivers expressed that the PD AI Chatbot had a positive impact on their experience. [24] |
|  |  | "I learned to manage my fistula and the different puncture techniques. It is no longer just the nurses' choice on how to puncture my fistula, it's my choice too." (Person on haemodialysis). [20] |
| **Implementation Process** | | |
|  | Teaming | N/A |
|  | Assessing needs | N/A |
|  | (1) Innovation deliverers |  |
|  | (2) Innovation recipients |  |
|  | Assessing context | N/A |
|  | Planning | N/A |
|  | Engaging | N/A |
|  | (1) Innovation deliverers |  |
|  | (2) Innovation recipients |  |
|  | Doing | - Conflicts with daily routine (-) |
|  |  | Other barriers to app use included limited time, forgetfulness and competing priorities, reflecting the difficulty of engaging this patient population in research. [25] |
|  |  | Impediments were mainly related to interference from work responsibilities, adverse effects of dialysis treatments (e.g., fistula complications, nausea, fatigue) and/or efforts to attend late‑night psychosocial support sessions. [20] |
|  |  | Fatigue was exacerbated by these busy lifestyles and, together with fluctuations in health and ability, this made scheduling physical activity challenging. [21] |
|  |  | Participants found that being able to use MK&M at a time that was convenient for them and working through the program at their own pace helped them to engage with it. [19] |
|  |  | - Poor portability of monitoring devices (-) |
|  |  | The kit was hard to carry around when traveling by plane. [23] |
|  | Tailoring strategies | N/A |
|  | Adapting | - Continuous content updates (+) |
|  |  | Other comments included ensuring the website was current. [26] |
|  |  | "Online knowledge of food with high potassium is not detailed and sometimes conflicting." (Patient 6, male, 34y, CKD G5 not dialysis; interviews). [27] |
|  |  | "For me, I already knew that kind of stuff, so I had a look at it and it was like, yeah, there's nothing really new here for me." (45-50 years, female, dialysis vintage 6-10 years). [25] |
|  |  | "I would say I'm quite well versed in matters relating to kidney disease at this point, so I don't know what [Kidney BEAM] could tell me that I kind of don't already know. " (Female, 20s, White British, haemodialysis, moderate adherence). [21] |
|  |  | Participants were hopeful that new information would continue to be released, with alerts and reminders to let them know when this was available. [19] |
|  | Reflecting & Evaluating | N/A |
|  | (1) Implementation |  |
|  | (2) Innovation |  |
