# Supplementary material for: Facilitators and Barriers to Digital Health Technologies for Self-Management in Patients With Chronic Kidney Disease: Systematic Review Based on the Updated CFIR 2.0 Framework
Source: J Med Internet Res. 2026 Sep 21;28:e103560. doi: 10.2196/103560 (PMC13592748; doi:10.2196/103560)
Supplement: Supplementary file 4 [file jmir-v28-e103560-s004.docx]

**List of excluded full-text articles with reasons for exclusion**

| No. | Title | Reason for exclusion |
| --- | --- | --- |
| 1 | Healthcare Providers' Perspectives on Implementing m-Health Interventions for Chronic Kidney Disease Care: A Qualitative Study | No association with self‑management |
| 2 | Movement through Active Personalised engagement (MAP) - a self-management programme designed to promote physical activity in people with multimorbidity: study protocol for a randomised controlled trial | No association with mobile health |
| 3 | Use of Wearable and Wireless Technology in Real-World Clinical Settings to Improve Patient Outcomes in Chronic Kidney Disease: a Mixed Methods Pilot Prospective Trial | No association with self‑management |
| 4 | Comments on “Perceptions of mHealth technology use by patients under dialytic treatment” – A Qualitative Perspective on Self-Management Support in Chronic Kidney Disease | Conference or letters or comments |
| 5 | Text Messaging Adherence Intervention for Adolescents and Young Adults with Chronic Kidney Disease: Pilot Randomized Controlled Trial and Stakeholder Interviews | Nonadult populations |
| 6 | Patients' Experiences and Perspectives of Telehealth Coaching with a Dietitian to Improve Diet Quality in Chronic Kidney Disease: A Qualitative Interview Study | No association with self‑management |
| 7 | Patients' and Nephrologists' Evaluation of Patient-Facing Smartphone Apps for CKD | No association with self‑management |
| 8 | Barriers and Enablers to m-Health Implementation in Nephrology Settings: A Qualitative Analysis of Clinician Experiences with Chronic Kidney Disease | No association with self‑management |
| 9 | Patient Experiences of Dietary Management in Chronic Kidney Disease: A Focus Group Study | No association with self‑management |
| 10 | Co-Designing Implementation Strategies for m-Health in Chronic Kidney Disease Care: A Qualitative Investigation | No association with self‑management |
| 11 | Readiness and service model exploration for chronic kidney disease telemonitoring at public hospital in Bengkulu, Indonesia | Conference or letters or comments |
| 12 | Implementation and Evaluation of a Patient-Focused eHealth Intervention, My Kidneys My Health, in Primary Care and General Nephrology Clinics: Multimethods Study | No association with self‑management |
| 13 | Home Telehealth: Facilitators, Barriers, and Impact of Nurse Support Among High-Risk Dialysis Patients | No association with self‑management |
| 14 | Randomised controlled trial of a smartphone application-based dietary self-management program on haemodialysis patients | No association with self‑management |
| 15 | Understanding the Gap Between m-Health Availability and Clinical Adoption in CKD Management: A Qualitative Study of Provider Readiness | No association with self‑management |
| 16 | Effect and Process Evaluation of e-Powered Parents, a Web-Based Support Program for Parents of Children With a Chronic Kidney Disease: Feasibility Randomized Controlled Trial | Nonadult populations |
| 17 | Perceptions of m-Health Technology Use Among Patients Undergoing Hemodialysis for Chronic Kidney Disease: A Qualitative Descriptive Study | No association with self‑management |
| 18 | Desirable Components for a Customized, Home-Based, Digital Care-Management App for Children and Young People With Long-Term, Chronic Conditions: A Qualitative Exploration | Nonadult populations |
| 19 | A Self-management Approach for Dietary Sodium Restriction in Patients With CKD: a Randomized Controlled Trial | No association with self‑management |
| 20 | Children and young people's views on access to a web-based application to support personal management of long-term conditions: a qualitative study | Nonadult populations |
| 21 | The combined diabetes and renal control trial (C-DIRECT) - a feasibility randomised controlled trial to evaluate outcomes in multi-morbid patients with diabetes and on dialysis using a mixed methods approach | No association with mobile health |
| 22 | Patients' Perspectives on m-Health for Symptom Monitoring in Chronic Kidney Disease: A Qualitative Interview Study | No association with self‑management |
| 23 | Randomized Control Trial on Internet-Based Self-Management of Arteriovenous Fistula in Hemodialysis Patients | Absence of full text |
| 24 | Optimising self-management in people living with chronic kidney disease (CKD) The Kidney Health 4 Life program | Absence of full text |
| 25 | A hospital-based lifestyle front office to provide patients guidance on lifestyle medicine-related issues | No association with mobile health |
| 26 | Feasibility of a Remotely Delivered Trial Testing an Online Self- Management Programme for People Living With CKD | Conference or letters or comments |
| 27 | Digital Self-Monitoring of Intake and Output Among Individuals With Kidney Failure: Awareness, Usability, Acceptance, Attitudes and Key Engagement Factors | No association with self‑management |
| 28 | 基于移动医疗APP的营养教育在腹膜透析患者中的应用 | No association with self‑management |
| 29 | Mobile Health Could Be a Solution in Overcoming Challenges of Care in Patients Who Need Peritoneal Dialysis | No association with self‑management |
| 30 | Perceived Facilitators and Barriers to Implementing a Technology Supported Primary Care Program for the Management of Type 2 Diabetes and Kidney Disease in India | Absence of full text |
| 31 | Kidney health literacy in the digital era: The experience of the Salud Renal Siempre platform | No association with self‑management |
| 32 | 互联网+血液透析通路全程化个案管理的应用效果研究 | No association with self‑management |
| 33 | 互联网+护理背景下中青年腹膜透析病人对居家透析治疗的认知与真实体验的质性研究 | No association with self‑management |
| 34 | 移动医疗结合情景模拟体验对慢性肾脏病2期患者远期疗效的影响 | No association with self‑management |
| 35 | The Online Parent Information and Support project, meeting parents' information and support needs for home-based management of childhood chronic kidney disease: research protocol | Nonadult populations |
| 36 | Patient Engagement with m-Health Content in Chronic Kidney Disease: A Qualitative Exploration of Feature Preferences | No association with self‑management |
| 37 | Design and evaluation of a mobile application to assist the self-monitoring of the chronic kidney disease in developing countries | No association with self‑management |
| 38 | PERCEPTIONS OF MHEALTH TECHNOLOGY USE BY PATIENTS UNDER DIALYTIC TREATMENT | No association with self‑management |
| 39 | Can Telehealth Improve Access to Dietary Management in Patients Receiving Dialysis? Insights from Consumers | No association with self‑management |
| 40 | Think-Aloud Evaluation of a Digital Health Intervention for Kidney Disease: Real-Time Qualitative Usability Testing | No association with self‑management |
| 41 | Virtual consultation in kidney care: a mixed-methods study on a model for safe and effective integration into routine clinical care | No association with self‑management |
| 42 | Usability Testing of the Kidney Score Platform to Enhance Communication About Kidney Disease in Primary Care Settings: Qualitative Think-Aloud Study | No association with self‑management |
| 43 | Attitudes and perceptions of patients on the kidney transplant waiting list toward mobile health–delivered physical activity programs | No association with self‑management |
| 44 | Supporting patients to be involved in decisions about their health and care: Development of a best practice health literacy App for Australian adults living with Chronic Kidney Disease | No association with self‑management |
| 45 | The Renal Health Instagram: An Analysis of Comments | Conference or letters or comments |
| 46 | [OP348 Assessing The Potential Value Of Wearable Digital Health Technologies In Chronic Kidney Disease Using Early Health Technology Assessment Methods](http://doi.org/10.1016/j.jval.2021.04.061) | Absence of full text |
| 47 | Patient Perspective of Smartphone-Based Apps for CKD Self-Care | Conference or letters or comments |
| 48 | Current and Emerging Applications of Digital Health for AKI | Conference or letters or comments |
| 49 | m-Health for Symptom Monitoring in Chronic Kidney Disease: A Qualitative Needs Assessment | No association with self‑management |
| 50 | Health literacy and guideline-adherent lifestyle in people with chronic kidney disease: exploring factors associated with usage intention of a structured m-health program and pilot data on actual behavior change | No association with self‑management |
| 51 | Feasibility and acceptability of telehealth coaching to promote healthy eating in chronic kidney disease: a mixed-methods process evaluation | No association with self‑management |
| 52 | Exploring How the Kidney BEAM Physical Activity Digital Health Intervention Improved Mental Health-Related Quality of Life for People Living With Kidney Disease | No association with self‑management |
| 53 | In-center hemodialysis patients' use of the internet in the United States: a national survey | No association with self‑management |
| 54 | An offline mobile nutrition monitoring intervention for varying-literacy patients receiving hemodialysis: a pilot study examining usage and usability | No association with self‑management |
| 55 | Qualitative Evaluation of Digital Symptom Management Tools in Advanced Chronic Kidney Disease: A Scoping Review | No association with self‑management |
| 56 | Dietary Application for the Management of Patients with Hemodialysis: A Formative Development Study | No association with self‑management |
| 57 | Development and Validation of an Application for Follow-up of Patients Undergoing Dialysis: NefroPortátil | No association with self‑management |
| 58 | Developing eHealth for Home Dialysis: Clinicians' Needs for a Digital Patient Engagement Platform | No association with self‑management |
| 59 | Designing a web-application to support home-based care of childhood CKD stages 3-5: qualitative study of family and professional preferences | Nonadult populations |
| 60 | A Coaching Program to Improve Dietary Intake of Patients with CKD: ENTICE-CKD | No association with self‑management |
| 61 | Using eHealth in the Continuity Care of Chronic Kidney Disease: Opportunities and Considerations | Absence of full text |
| 62 | User-centred design for developing e-Health system for renal patients at home (AppNephro) | Absence of full text |
| 63 | Readiness of patients with chronic diseases to use health information technology: An experience of a developing country | No association with self‑management |
| 64 | PUK21 PatientsÂ’ Preferences for a Wearable Digital Health Technology to Support Self-Management of Chronic Kidney Disease | Conference or letters or comments |
| 65 | POS-479 ACCEPTABILITY AND FEASIBILITY OF A WEB-BASED SELF-MANAGEMENT SUPPORT INTERVENTION FOR ADULTS WITH CHRONIC KIDNEY DISEASE: A MIXED-METHODS STUDY | Conference or letters or comments |
| 66 | Patient Perspectives on m-Health for Physical Activity and Symptom Management in CKD: A Qualitative Systematic Review | No association with self‑management |
| 67 | User Feedback on Digital Symptom Trackers in Chronic Kidney Disease Care: A Qualitative Interview Study | No association with self‑management |
| 68 | WCN24-1842 NEPHROLOGISTS PERSPECTIVES AND ATTITUDES ON THE USE OF TELEHEALTH IN MANAGING PATIENTS WITH CHRONIC KIDNEY DISEASE | Conference or letters or comments |
| 69 | TeleClinical-Care for Chronic Kidney Disease: Acceptability and Initial User Testing of a Virtual Delivery Platform...Renal Society of Australasia Conference, May 29-31, 2023, Sydney, New South Wales | No association with self‑management |
| 70 | Mobile Health (mHealth) Technology: Assessment of Availability, Acceptability, and Use in CKD | Absence of full text |
| 71 | Self-Management Intervention through Lifestyle Education for Kidney health' (the SMILE-K study): protocol for a single-blind longitudinal randomised controlled trial with nested pilot study | No association with mobile health |
| 72 | Results of a pilot feasibility randomised controlled trial exploring the use of an electronic patient-reported outcome measure in the management of UK patients with advanced chronic kidney disease | No association with self‑management |
